# Methylosome and survival motor neuron complexes are dispensable for plant viability in *Arabidopsis thaliana*

**DOI:** 10.1093/plphys/kiag667

**Published:** 2026-09-05

**Authors:** Daniela Goretti, Silvio Collani, Sarah Muniz Nardeli, Hemamshu Ratnakaram, Stéphanie Robert, Markus Schmid

**Affiliations:** Department of Plant Physiology, Umeå Plant Science Centre, Umeå University, Linneus väg 6, Umea SE-90187, Sweden; Council for Agricultural Research and Economics – Research Centre for Forestry and Wood (CREA-FL), Strada Frassineto 35, 15033 Casale Monferrato, AL, Italy; Department of Plant Physiology, Umeå Plant Science Centre, Umeå University, Linneus väg 6, Umea SE-90187, Sweden; DISSTE, University of Eastern Piedmont, Piazza S. Eusebio 5, 13100 Vercelli, Italy; Department of Plant Physiology, Umeå Plant Science Centre, Umeå University, Linneus väg 6, Umea SE-90187, Sweden; Department of Plant Biology, Linnean Center for Plant Biology, Swedish University of Agricultural Sciences, Almas allé 5, Uppsala SE-75007, Sweden; Department of Forest Genetics and Plant Physiology, Umeå Plant Science Centre, Swedish University of Agricultural Sciences, Lineeus väg 6, Umea SE-90183, Sweden; Department of Forest Genetics and Plant Physiology, Umeå Plant Science Centre, Swedish University of Agricultural Sciences, Lineeus väg 6, Umea SE-90183, Sweden; Department of Plant Physiology, Umeå Plant Science Centre, Umeå University, Linneus väg 6, Umea SE-90187, Sweden; Department of Plant Biology, Linnean Center for Plant Biology, Swedish University of Agricultural Sciences, Almas allé 5, Uppsala SE-75007, Sweden

## Abstract

The role of RNA splicing as a modulator of the molecular responses to stress is well described. In contrast, its importance in the acclimation of plants to changes in ambient temperatures has only recently started to emerge. Here, we analyzed the role of temperature in regulating the functionality of factors associated with small nuclear ribonucleoprotein (snRNP) biogenesis, a key process underlying pre-mRNA splicing. Taking advantage of mutants showing temperature-dependent phenotypes, we conducted a comprehensive study of the role that the methylosome and survival motor neuron (SMN) complexes have in plant development. Genetic, phylogenetic, and confocal analyses, as well as in vivo and in vitro evidence, reveal remarkable differences in the composition and importance of these complexes between plants and vertebrate animals. The SMN complex in Arabidopsis is apparently reduced to a single protein, GEMIN2, that is not essential for plant development, and the existence of an SMN ortholog is uncertain. Similarly, components of the methylosome previously implicated in snRNP biogenesis are not essential for plant viability. Our results suggest that factors considered central to snRNP biogenesis in animals have less crucial roles in plants and highlight how an evolutionarily conserved molecular process like RNA splicing has nevertheless evolved plant-specific characteristics.

## Introduction

RNA splicing is an essential regulatory mechanism occurring in all eukaryotic organisms. The splicing reaction, during which introns are removed from the primary transcript and exons are joined, is catalyzed by large ribonucleoprotein complexes, the spliceosomes (Wahl et al. 2009). At the heart of each spliceosome are 7 Sm proteins, called B/B′, D1, D2, D3, E, F, and G, that form a heptameric ring into which different U-rich small nuclear RNAs (snRNAs), U1, U2, U4, and U5, are incorporated, after which the resulting small nuclear ribonucleoprotein (snRNP) complexes are named. In addition, splicing requires a fifth snRNP that consists of a ring of 7 Sm-like (LSm) proteins, LSm2–LSm8, and the U6 snRNA. An alternative heptameric LSm ring, consisting of LSm1–LSm7, is involved in cytoplasmic nonsense-mediated RNA decay (Mayes et al. 1999; He and Parker 2000; Golisz et al. 2013).

Importantly, in animals the assembly of the Sm and LSm rings involves different mechanisms. LSm proteins have the inherent capacity to self-assemble into ring-shaped multiprotein complexes, binding snRNAs only in a second step (Achsel et al. 1999). In contrast, Sm proteins, although originating from a duplication of LSm genes, have lost their ability to self-assemble in vivo and are assembled in a step-wise process (Friesen et al. 2001; Meister and Fischer 2002; Veretnik et al. 2009).

In vertebrate animals, the assembly of the Sm ring in the cytoplasm requires 2 protein complexes referred to as methylosome and survival motor neuron (SMN) complex (Matera and Wang 2014). The human methylosome consists of the protein arginine methyltransferase 5 (PRMT5) and methylosome protein 50 (MEP50/WP45) (Antonysamy et al. 2012). These core components bind to chloride channel nucleotide sensitive 1A (PICLN/CLNS1A), which acts as molecular chaperone, to facilitate methylation of several Sm proteins (Pesiridis et al. 2009). Specifically, PRMT5 is a type II arginine methyltransferase that catalyzes the symmetric di-methylation (SDM) of RG motives in the C-terminal region of the B/B′, D1, and D3 Sm proteins (Bedford and Richard 2005; Bedford and Clarke 2009). This posttranslational modification increases the affinity of these subunits to the SMN complex, facilitating the next step of snRNP assembly. Interestingly, in *Drosophila melanogaster*, which belongs to the order Diptera, methylation of Sm proteins by Dart5, the PRMT5 ortholog in fruit flies, is dispensable for snRNP biogenesis, indicating that although the process of RNA splicing is shared across the eukaryotic kingdoms and the Sm proteins and snRNAs are highly conserved, the requirement for Sm protein methylation has changed among taxa throughout evolution (Gonsalvez et al. 2008; Kroiss et al. 2008).

Similar to the methylosome, also the SMN complex differs between taxa. The human SMN complex includes the SMN protein (encoded by the duplicated *SMN1* and *SMN2* genes) and 6 GEMIN proteins (GEMIN2–GEMIN7) with different molecular functions (Gubitz et al. 2004). The SMN complex brings together the intermediate SmD1–SmD2 and SmB–SmD3 heterodimers and the SmF–SmE–SmG heterotrimer and assembles them all around a specific snRNA molecule, providing binding specificity to avoid Sm proteins from binding illicit RNAs (Pellizzoni et al. 2002). Upon interaction with import factors, the entire complex translocates into the nucleus, where eventually the SMN complex disassembles, exerting other nucleus-specific functions, and the snRNP biogenesis is completed (Matera and Wang 2014; Gruss et al. 2017). Even within the metazoan clade, the large SMN complex found in humans, and vertebrates in general, is considerably simplified in other orders. For example, in *D. melanogaster* the SMN complex consists of only GEMIN2 and SMN (Gonsalvez et al. 2008; Kroiss et al. 2008). Similar differences have been noted between different classes of fungi, with *Schizosaccharomyces pombe* requiring SMN and GEMIN2-6-7-8 and the support of PICLN for snRNP assembly. Interestingly, in *Saccharomyces cerevisiae*, this process has been shown to be reduced to even fewer components, involving only the GEMIN2 ortholog BRR1P and the PICLN ortholog LOT5 (Kroiss et al. 2008; Hu et al. 2023; Wang et al. 2025). More recently, it was demonstrated that the Sm ring can even assemble spontaneously without the need for BRR1P and LOT5 (Wang et al. 2025). Supporting the theory of self-assembly of the Sm ring in yeast, a role for arginine methylation in snRNP biogenesis and RNA splicing has not been demonstrated (Low and Wilkins 2012; Hamey and Wilkins 2023). However, BRR1P is required for vegetative growth of *S. cerevisiae* under specific perturbations, such as weak *sm* mutant alleles (Schwer et al. 2017).

In plants, phylogenetic analyses suggest that the SMN complex may resemble the minimal complex found in *Drosophila*, consisting of GEMIN2 and SMN, although the role of the candidate plant SMN protein in snRNP assembly and splicing has not yet been experimentally validated (Kroiss et al. 2008). More recently, phylogenetic analyses that account for specific features of the SMN glycine zipper motif (YG box) have suggested the existence of SMN-like proteins in certain plant groups or species (Gupta et al. 2021). Importantly, no orthologs of these candidate SMN-like proteins were identified in *Arabidopsis thaliana* (Gupta et al. 2021).

The uncertainty surrounding the identity of SMN in plants is mirrored by the methylosome, which lacks a MEP50 ortholog and whose role in snRNP assembly remains unclear (Kroiss et al. 2008; Mateos et al. 2023). The hypothesis that the plant methylosome consists solely of PICLN and PRMT5 is supported by the finding that, in Arabidopsis, PRMT5 directly interacts with PICLN and Sm proteins, as well as by the lethality of *picln prmt5* double mutants (Huang et al. 2016; Mateos et al. 2023).

An important aspect of RNA splicing conserved across eukaryotes is its modulation by environmental factors, such as temperature. Indeed, organisms ranging from humans to plants can sense changes in temperature and integrate this information at the molecular level through alternative pre-mRNA splicing (AS) (Bartok et al. 2013; Dikaya et al. 2021). The selection of splice acceptor and donor sites during AS of a particular transcript involves multiple processes, including chromatin remodeling, regulation of the transcriptional machinery and transcriptional kinetics, and posttranslational modifications of splicing factors (Kornblihtt et al. 2013). More recently, particularly in plants, a core component of the spliceosome, SmE1/PORCUPINE (PCP), as well as members of the snRNP assembly machinery, PICLN and GEMIN2, have been reported to modulate AS in response to temperature changes (Schlaen et al. 2015; Capovilla et al. 2018; Huertas et al. 2019; Mateos et al. 2023; El Arbi et al. 2024; Goretti et al. 2025).

Importantly, whereas loss-of-function mutants of *SMN*, *GEMINs*, *PICLN*, and *PRMT5* are lethal in mice, the corresponding single mutants are viable in plants (Jablonka et al. 2000; Pu et al. 2000; Monani 2005; Tee et al. 2010). Here, we took advantage of these viable mutants and the fact that their phenotypes are modulated by ambient temperature to explore the function of genes putatively associated with snRNP biogenesis in Arabidopsis and their role in plant growth and development. Overall, our analyses support the hypothesis that the SMN complex in Arabidopsis consists exclusively of GEMIN2 and that methylation of Sm proteins plays only a minor role in plant growth and development across a wide range of ambient temperatures. This is consistent with the situation in *S. cerevisiae* and supports the existence of alternative mechanisms for the assembly of the heptameric Sm ring in plants.

## Results

### The methylosome is dispensable for survival but required for plant growth and development

**Figure 1:**
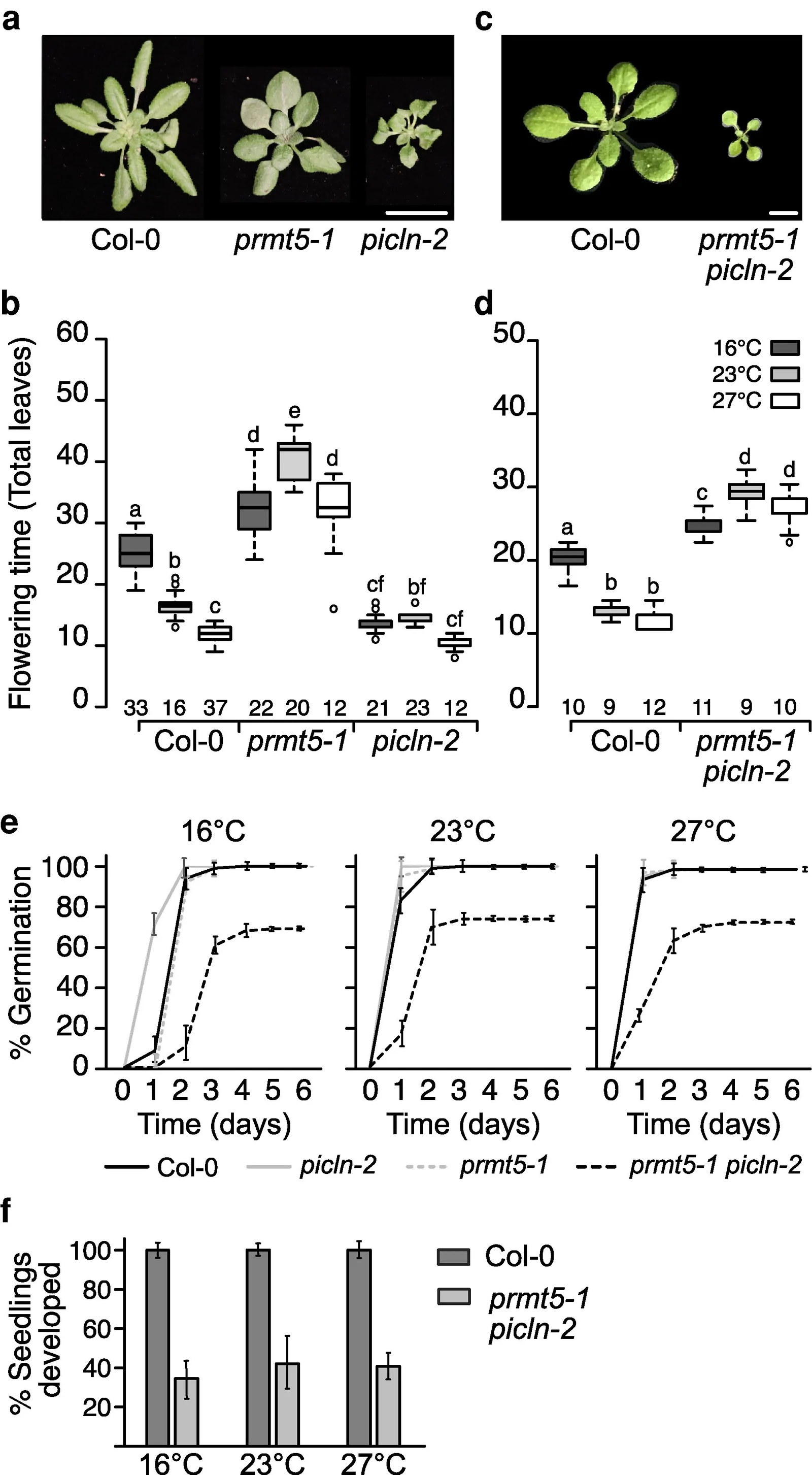
The methylosome is dispensable for plants growing at ambient temperature. a) Phenotypes of Col-0, *prmt5-1*, and *picln-2* grown in LD at 16 °C for 33 d. Scale bar: 1 cm. b) Flowering time of the 3 genotypes in (a) at 3 different temperatures. Numbers on the *x* axis indicate the number of individuals per genotype and condition. One-way ANOVA was used for statistical analysis. Different letters indicate categories that are statistically different after Tukey–Kramer correction (*P* ≤ 0.05). c) Phenotype of Col-0 and the *prmt5-1 picln-2* double mutant grown at 23 °C for 20 d after sowing. Scale bar: 0.5 cm. d) Flowering time of the 2 genotypes in (c) at 3 different temperatures. Numbers on the *x* axis indicate the number of individuals. One-way ANOVA was used for statistical analysis. Different letters indicate categories that are statistically different (*P* ≤ 0.05). e) Germination rate of Col-0, *picln-2*, *prmt5-1*, and *prmt5-1 picln-2* seeds on MS plates at 3 different temperatures. f) Percentage of germinated Col-0 and *prmt5-1 picln-2* seeds developing into young plants at 16, 23, and 27 °C. E, F, *prmt5-1 picln-2* seeds used were harvested from double mutant plants grown at 27 °C; Col-0 and single mutant seeds were harvested from plants grown at 23 °C. Data show the results from 3 independent replicates per genotype and temperature. Error bars indicate standard deviation.

As widely described in metazoans, the snRNP assembly begins with methylation of SmD1, SmD3, and SmB by the methylosome. In Arabidopsis, 2 proteins, PRMT5 and PICLN, are reported to form the methylosome and have been implicated in plant responses to abiotic stresses, such as salt and cold (Hu et al. 2017; Mateos et al. 2023). Given that the growth of *Sm* and *GEMIN2* mutants is affected by temperature (Schlaen et al. 2015; Capovilla et al. 2018; Nardeli et al. 2023), we assessed the role of *PRMT5* and *PICLN* in acclimation to low and high ambient temperatures by growing mutants under control conditions (23 °C) and at 16 and 27 °C, which represent the lower and upper limits of the ambient temperature range, beyond which Arabidopsis experiences cold stress or heat stress, respectively (Guo et al. 2018; Hayes et al. 2021). We found that the *picln* and *prmt5* null mutants exhibit opposing flowering time phenotypes (Figure S1a). *prmt5-1* (and *prmt5-2*) flowered consistently later than Col-0 at all temperatures tested, whereas *picln-2* was early flowering at 16 °C but flowered similarly to wild type at 23 and 27 °C (Fig. 1a, b; Figure S1b, c). *picln* mutants showed growth defects, particularly at 16 °C, and were overall smaller than wild type at all tested temperatures (Figure S1d, e; Table S1). Furthermore, expression of both *PICLN* and *PRMT5* was moderately induced at 16 °C when compared to 23 and 27 °C, in contrast to *GEMIN2*, which was stably expressed across the examined temperature range (Figure S1f).

The opposing phenotypes of *picln* and *prmt5* mutants under the tested conditions prompted us to test the hypothesis that other PRMT family proteins could be components of the plant methylosome. In particular, we focused on PRMT7/PRMT16, as the human homolog has previously been shown to interact with SmD3 and SmB (Gonsalvez et al. 2007; Zurita-Lopez et al. 2012). However, we were unable to detect interactions between AtPRMT7 and PICLN or any of the Sm proteins tested using the heterologous yeast 2-hybrid (Y2H) system (Figure S2a). Furthermore, under the tested conditions, the *prmt7-1* null mutant showed no discernible phenotype (Figure S2b, c). This suggests that PRMT5 is the main arginine methylase capable of directly interacting with the splicing machinery in plants. This is consistent with a previous report by Mateos et al. (2023), which showed that PRMT5 interacts directly with PICLN and demonstrated that the double mutant is embryo lethal, indicating genetic interaction between the 2 genes, as also suggested by Huang et al. (2016). However, our genetic analysis did not indicate an epistatic interaction between PRMT5 and PICLN, suggesting that double mutants are not necessarily lethal.

Indeed, we succeeded in establishing the *prmt5-1 picln-2* double mutant, provided that the parental plants used for the cross (*F*_0_) and subsequent generations (*F*_1_; *F*_2_) were grown at 27 °C, a condition under which the *picln-2* phenotype is largely reverted to normal. Interestingly, the progeny of *prmt5-1 picln-2* double mutants propagated at 27 °C, although generally smaller than the single mutants and Col-0 controls, were viable and set seeds even when grown at lower temperatures (Fig. 1c, d; Figure S1g). In addition, the germination rate of the double mutant was reduced by approximately 30% compared to the single mutants and Col-0 (Fig. 1e). Development of 60% of the *prmt5-1 picln-2* seedlings arrested shortly after germination, and these plants failed to form proper leaves (Fig. 1f), indicating that the previously reported lethality of the *prmt5-1 picln-2* double mutant is a moderately penetrant phenotype.

Taken together, the mutant phenotypes and genetic analyses, in particular the viability of the *prmt5-1 picln-2* double mutant, suggest that the 2 known members of the methylosome might play a less prominent role in plant survival across the ambient temperature range than previously thought.

### SDM of arginine residues at the C-terminus of SmD1b is dispensable at ambient temperature

It is well established that methylation of SmD1, SmD3, and SmB by the methylosome supports snRNP assembly via the SMN complex in humans (Matera and Wang 2014). However, the observation that the *prmt5-1 picln-2* double mutant is viable provides indirect evidence that the PICLN–PRMT5 methylosome is dispensable for plant development. We therefore investigated the importance of SDM for Sm protein and function more directly, using SmD1b as an example.

To gain insight into the role of SDM of SmD1b in function in plants, we cloned a truncated version of the SmD1b open reading frame lacking the last 16 amino acids (hereafter SmD1bΔC), as well as a construct in which the last 8 arginine residues in the C-terminal region of SmD1b, which constitute potential targets for SDM by PRMT5 (Brahms et al. 2001), were mutated to lysine (hereafter SmD1b^Lys^) (Figure S3a). It should be noted that in humans, the equivalent single RG motive at position 61, which is retained in our constructs, has been shown not to be involved in the interaction between methyltransferases and SNRPD1 (Friesen et al. 2001). Therefore, this RG motif was excluded from our mutagenesis strategy.

**Figure 2:**
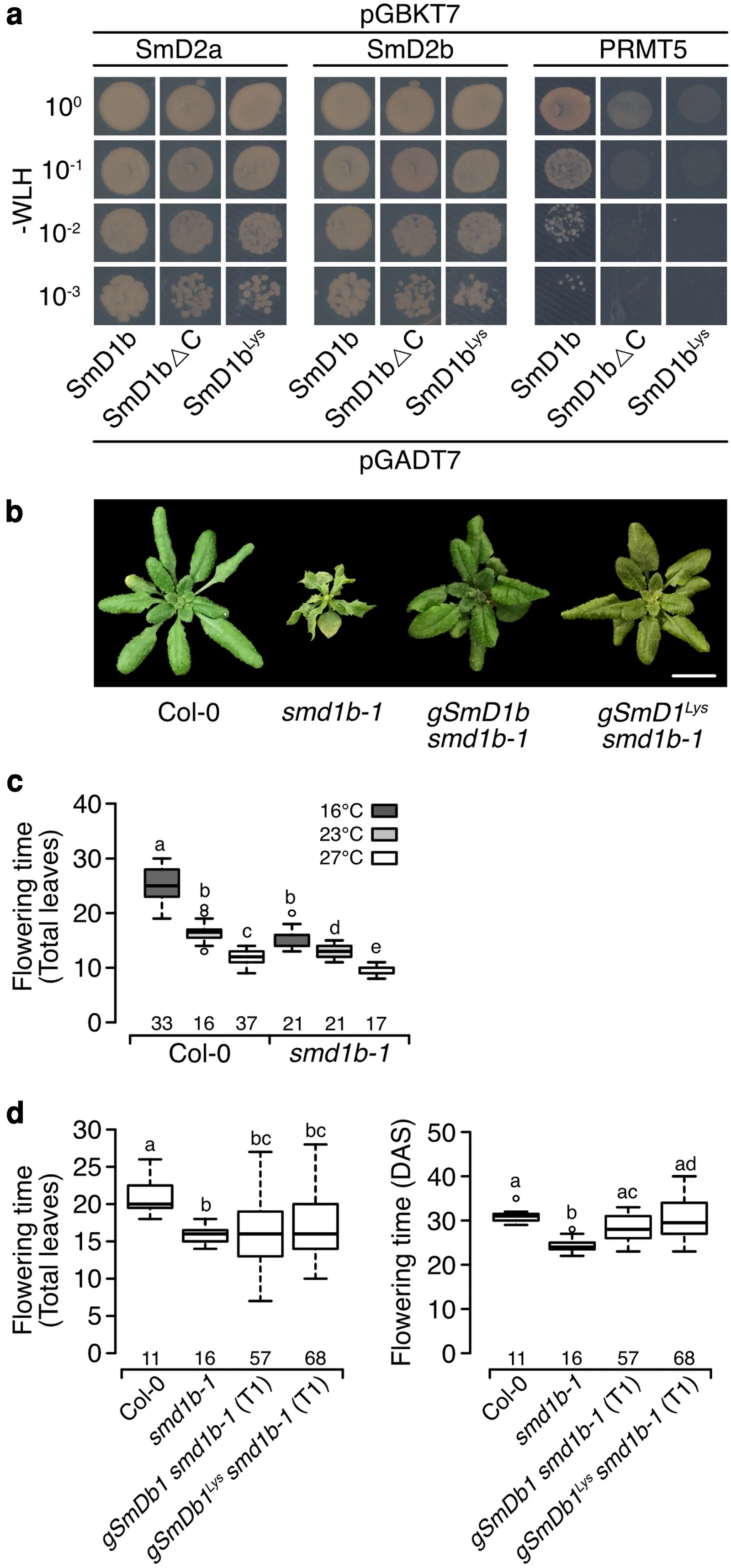
SDM of SmD1b is dispensable at ambient temperatures. a) Interaction between unmodified SmD1b, SmD1b lacking the C-terminal 16 amino acids (ΔC), and SmD1b in which 8 arginine residues in the C-terminal half have been replaced by lysine (Lys) with SmD2 and PRMT5. pGADT7 and pGBKT7 contribute the AD and BD domains, respectively. Photos were taken after 4 d of growth on selective medium. b) Phenotype of Col-0, *smd1b-1*, and T1 *smd1b-1* plants complemented with either wild-type (gSmD1b) or a protein version in which the last 8 arginine residues have been replaced by lysine (gSmD1b^Lys^) grown at 16 °C for 30 d. Scale bar: 1 cm. c) Flowering time of Col-0 and *smd1b-1* mutant at 3 different temperatures. d) Flowering time of Col-0, *smd1b-1*, and independent T1 transformants carrying either the wild-type (*n* = 57) or mutagenized (*n* = 68) version of *gSmD1b* grown at 16 °C. DAS, days after sowing. Numbers on the *x* axis indicate the number of individuals per genotype and condition. One-way ANOVA was used for statistical analysis. Different letters indicate categories that are statistically different after Tukey–Kramer correction (*P* ≤ 0.05).

Y2H analyses confirmed that PRMT5 interacts with wild-type SmD1b, as previously shown by Mateos et al. (2023). This interaction was abolished in SmD1bΔC and SmD1b^Lys^, indicating that the C-terminal arginine residues of SmD1b were essential for the interaction with PRMT5 and suggesting that the remaining single RG motif at position 61 was indeed not sufficient to mediate this interaction (Fig. 2a; Figure S3b). In contrast, deletion or mutation of the C-terminal region had only a negligible effect on the interaction with SmD2a and SmD2b, which, although encoded by different genes, share an identical amino acid sequence and interact with SmD1b to form one of the heterodimers from which snRNPs are assembled (Zhang et al. 2011) (Fig. 2a; Figure S3b). Taken together, these results suggest that SDM of SmD1b may not be required for snRNP assembly in plants.

To test this hypothesis, we took advantage of the *smd1b-1* null mutant, which displays developmental defects under normal growth conditions (Elvira-Matelot et al. 2016). Similar to the other mutants discussed, we found that the pleiotropic phenotype of *smd1b-1* was enhanced at lower ambient temperatures (Fig. 2b; Figure S3c). At 16 °C, *smd1b-1* plants flowered early and showed altered leaf morphology and smaller rosette diameter compared to wild type, similar to the *picln-2* and *gemin2-2,* a knockout mutant of a protein putatively involved in Sm ring assembly and described in the following paragraphs (Fig. 2b, c; Figure S3c, d; Table S1).

Next, we expressed the wild-type SmD1b and the mutagenized SmD1b^Lys^ open reading frame under the control of the *35S* promoter or a 1,578 bp *SmD1b* promoter fragment in the *smd1b-1* background. Phenotypes were assessed in T1 plants grown at 16 °C, a condition under which the mutant exhibits a strong phenotype. While the expression of the 2 proteins under the endogenous *SmD1b* promoter was not able to complement the *smd1b-1* phenotype (>40 T1 plants), the use of the constitutive *35S* promoter resulted in full complementation, indicating that the rescue construct using the *SmD1b* promoter was lacking important cis-regulatory elements (Figure S3e, f).

To overcome this problem, we generated a genomic *SmD1b* wild-type rescue construct (*gSmD1b*) consisting of the 1,578 bp promoter, all exons, introns and a 2,003bp terminator. We then mutated the last 8 arginine residues in the C-terminal region of SmD1b in this construct, generating gSmD1b^Lys^. Both the wild-type and *gSmD1b^Lys^* genomic constructs complemented the reduced size and early flowering phenotype of the *smd1b-1* mutant in the majority of independently transformed T1 plants (Fig. 2d; Figure S4a, b). However, only *gSmD1B* restored the full wild-type phenotype in 91% of T1 plants (52/57), exhibiting a significantly higher rescue efficiency compared to *gSmD1b^Lys^* (68%, 22/69; *P* < 0.001, 2-tailed Fisher’s exact test, odd ratio = 5.67) (Figure S4a, b).

The finding that a variant of SmD1b, SmD1b^Lys^, unable to interact with PRMT5, and therefore unlikely to be methylated, largely retains functionality suggests that Sm protein methylation is not essential in Arabidopsis, raising the possibility that the molecular mechanism controlling snRNP assembly differs between Arabidopsis and humans.

### Arabidopsis lacks an obvious ortholog of the human SMN protein

Having established that SDM is likely dispensable for SmD1b function in plants, we next focused on the SMN complex, which orchestrates the subsequent step in the assembly of the heptameric Sm ring. Whether plants encode an ortholog of the human SMN protein remains a matter of debate, as previous phylogenetic analyses were inconclusive (Kroiss et al. 2008; Schlaen et al. 2015). We therefore investigated *AtSPF30* (*SPLICING FACTOR 30*, At2g02570), which has been proposed as a potential Arabidopsis SMN homolog, using genetic and molecular approaches (Kroiss et al. 2008; Schlaen et al. 2015).

**Figure 3:**
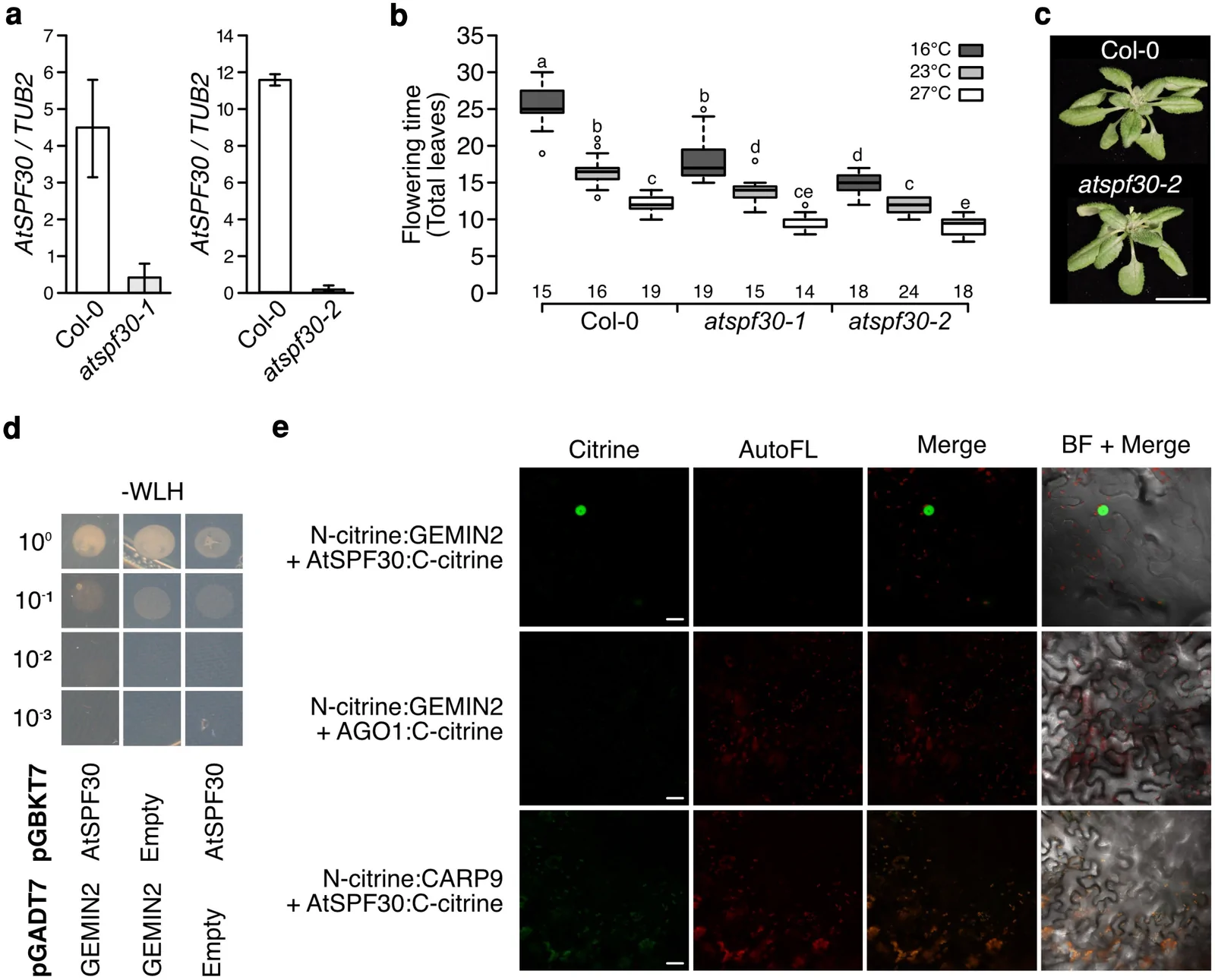
AtSPF30 lacks the properties expected of a canonical SMN ortholog. a) Expression of *AtSPF30* in Col-0 and 2 *atspf30* mutants. b) Flowering time of 2 *atspf30* alleles and control plants grown in LD at 3 different temperatures. Numbers on the *x* axis indicate the number of individuals per condition. One-way ANOVA was used for statistical analysis. Different letters indicate categories that are statistically different after Tukey–Kramer correction (*P* ≤ 0.05). c) Phenotype of 33-day-old *atspf30-2* and Col-0 grown in LD at 16 °C. d) Interaction between GEMIN2 and AtSPF30. pGADT7 and pGBKT7 contribute the AD and BD domains, respectively. Photos were taken after 4 d of growth on selective drop-out medium. e) BiFC analysis of GEMIN2 and AtSPF30 interaction in transiently transformed tobacco leaves. CARP9 and AGO1 were used as negative controls. All 3 biological replicates for CARP9-AtSPF30 are shown in Figure S5d. The BiFC signal was detected 48 h after infiltration by confocal microscopy. Scale bar: 20 *μ*m.

First, we characterized 2 available mutant alleles, *atspf30-1* (knockdown) and *atspf30-2* (knockout), under different temperature conditions (Kroiss et al. 2008; Romanowski et al. 2020) (Fig. 3a). We observed that *atspf30* mutants flowered earlier than wild type, particularly at lower ambient temperature (Fig. 3b; Figure S5a). Apart from the early flowering phenotype, *atspf30* mutants did not display any major phenotypic defects (Fig. 3c). Next, we tested whether AtSPF30 interacted with GEMIN2 in a Y2H assay, as is the case for the human SMN and GEMIN2 proteins. However, no yeast growth was detected on selective media, indicating the absence of a detectable interaction (Fig. 3d; Figure S5b). To validate the assay, we included positive controls. Both 6B-interacting protein 1-like-1 (ASIL1, At1g54060) and SmGb interacted with AtSPF30 and GEMIN2, respectively, confirming that the assay functioned as expected (Figure S5b, c). In contrast, we detected an interaction between GEMIN2 and AtSPF30 using bimolecular fluorescence complementation (BiFC) following transient expression in tobacco leaves (Fig. 3e; Figure S5d). Negative controls were performed in triplicate for both proteins. A fluorescent signal was observed in one biological replicate for the negative control combining N-Citrine:CARP9 (CONSTITUTIVE ALTERATIONS IN THE SMALL RNA PATHWAYS9, At4g13170) with AtSPF30:C-Citrine. However, this signal was weaker than that observed for the tested interaction and displayed a different subcellular localization, suggesting that it likely represented a false-positive event (Figure S5d).

The absence of a strong phenotype in *atspf30* mutants, together with the different behavior of GEMIN2 and AtSPF30 in yeast and plant cells, supports the hypothesis that AtSPF30 might not be the ortholog of the human SMN protein, as previously suggested. In fact, in metazoans SMN mutants are lethal and the interaction between GEMIN2 and SMN is strong and direct (Kroiss et al. 2008).

To continue the search for a potential plant ortholog of human SMN, we performed additional phylogenetic and BLAST analysis. Neither BLASTP searches within the embryophyte clade nor ortholog prediction based on human SMN1 sequence identified convincing plant candidates. However, domain-based analysis using DELTA-BLAST detected multiple potential SMN-related proteins across diverse plant species (Figure S6a; Table S2). In Arabidopsis, the analysis identified TUDOR-SN1 (TSN1, At5g07350) as possible candidate, although the similarity was limited to the Tudor domain shared by TSN1 and human SMN1 (Figure S6b). In Arabidopsis, *TSN1* and *TSN2* encode for two 84% identical proteins. The single mutants are indistinguishable from wild type, whereas the double mutant grows normally under ambient conditions but suffers severe defects in seed germination, seedling growth, and survival during high salinity stress (dit Frey et al. 2010).

This observation is in sharp contrast with the expected phenotype of an SMN1 ortholog, given the severe developmental defects and lethality associated with SMN dysfunction in metazoans. The absence of such a strong phenotype in *tsn* mutants, together with the limited sequence similarity between SMN1 and TSN1, which is confined to the Tudor domain, and the finding that TSN1 lacks obvious GEMIN2-binding, proline-rich and YG regions required for SMN1 function in humans, led us to exclude TSN1 from further investigations.

Finally, to identify proteins that might fulfill the role of SMN during snRNP assembly in plants, we performed a Y2H screen using Arabidopsis GEMIN2 as bait. Although several GEMIN2-interacting proteins were identified (Table S3), none possessed the characteristic features of human SMN, including an N-terminal GEMIN2-binding domain, a TUDOR domain capable of binding Sm proteins, or a C-terminal YG box required for SMN–GEMIN2 interaction.

As neither in silico nor in vitro approaches identified a clear SMN ortholog in Arabidopsis, we speculate that plants may either possess an alternative SMN complex with a distinct composition, potentially including proteins with divergent properties that have not yet been described in other taxa, or that, similar to the situation in yeast, plant snRNPs can self-assemble.

### Ambient temperature modulates the effects of *GEMIN2* on plant growth

Because the human SMN complex is a large multiprotein assembly, we performed phylogenetic analyses to identify potential orthologs of other GEMIN proteins in plants. However, neither the phylogenetic analyses nor our Y2H screen provided evidence for the existence of plant orthologs of GEMIN proteins other than GEMIN2 (Figure S7).

In Arabidopsis, *GEMIN2* has been shown to affect pre-mRNA splicing in response to cold treatment and to be required for normal plant growth at 10 °C (Schlaen et al. 2015). Defects in splicing have also been linked to growth abnormalities in *gemin2* mutants grown at 22 °C (Schlaen et al. 2015). To further characterize the role of GEMIN2 in ambient temperature responses, we grew the *gemin2-2* mutant at temperatures ranging from 16 to 27 °C.

**Figure 4:**
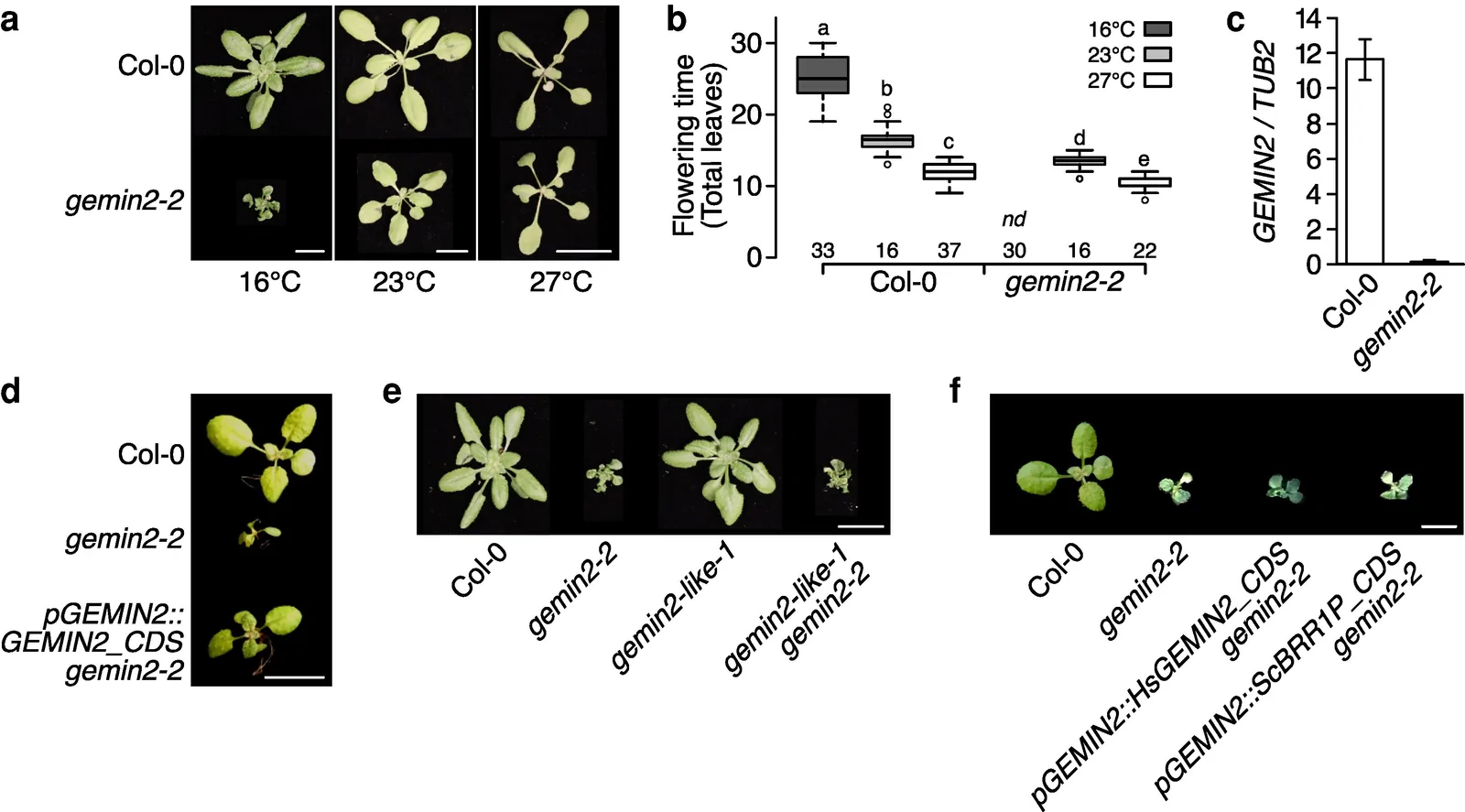
*Gemin2* phenotype is modulated by ambient temperature. a) Phenotype of *gemin2-2* and Col-0 grown in LD at different temperatures. Photos were taken 18, 25, and 33 d after sowing for plants grown at 27, 23, and 16 °C, respectively. b) Flowering time of Col-0 and *gemin2-2* grown in LD at 3 different temperatures. Numbers on the *x* axis indicate the number of individuals. One-way ANOVA was used for statistical analysis. Different letters indicate categories that are statistically different after Tukey–Kramer correction (*P* ≤ 0.05). c) Expression of *GEMIN2* in wild-type and mutant background. d) Rescue of the *gemin2-2* mutant by a *pGEMIN2::GEMIN2CDS* transgene. Plants were grown at 16 °C in LD and photos were taken 3 wk after sowing. e) Comparison of the rosette phenotype of Col-0, *gemin2-2*, *gemin2-like-1*, and *gemin2-2 gemin2-like-1* double mutants grown in LD at 16 °C for 33 d. Scale bars: 1 cm. f) Phenotype of Col-0, *gemin2-2*, and of *gemin2-2* complemented with human (Hs) and yeast (Sc) GEMIN2 orthologs expressed from the Arabidopsis *GEMIN2* promoter at 16 °C. Scale bar: 0.5 cm.

At 23 °C, the *gemin2-2* null mutant exhibited pleiotropic developmental defects, including early flowering, reduced rosette size, decreased leaf curvature, altered leaf morphology, and short petioles (Fig. 4a–c; Figure S8a–c; Table S1). All these phenotypes were strongly enhanced when the mutant was grown at low ambient temperature (16 °C) (Fig. 4a, b; Figure S8a–c; Table S1). Under this condition, *gemin2-2* plants displayed a bushy growth habit with crumpled and irregularly arranged leaves, in contrast to the regular spiral phyllotaxis observed in the wild type. Plants also remained severely stunted throughout their entire life cycle, with a rosette diameter of less than 2 cm even 70 d after sowing, when they eventually bolted and produced siliques (Table S1). Importantly, the severe phenotype observed in *gemin2-2* at 16 °C was largely rescued by expression of the *GEMIN2* coding sequence (CDS) under the control of its native promoter (*pGEMIN2::GEMIN2CDS)*, confirming that loss of *GEMIN2* was responsible for the observed growth defects (Fig. 4d). Similarly, several developmental defects of *gemin2-2* were partially rescued at elevated ambient temperature (27 °C) (Fig. 4a, b; Table S1). Importantly, the role of temperature in regulating the phenotype of *gemin2-2*, *picln-2*, and *prmt5-1* was statistically validated by a 2-way ANOVA that showed a significant interaction effect (*P* < 0.001), suggesting that genotype and temperature combine to influence the phenotype.

Phylogenetic analysis identified At2g42510, hereafter referred to as *GEMIN2-like*, as a likely paralog of *GEMIN2* in Arabidopsis. Consistent with this, both proteins are annotated as SMN-interacting proteins and contain a SIP1 (SMN-Interacting Protein 1) domain (Figure S7a). We therefore examined the performance of *gemin2-*like mutants across different ambient temperatures. Notably, a knockout *gemin2-like* T-DNA insertion mutant, *gemin2-like-1*, did not display any of the phenotypes observed in *gemin2-2* and was essentially indistinguishable from wild type under all tested conditions (Fig. 4e; Figure S8b–e). To assess potential additive or epistatic interactions between the 2 genes, we generated a *gemin2-2 gemin2-like* double mutant. Regardless of growth conditions, the double mutant always resembled the *gemin2-2* single mutant, indicating the absence of detectable genetic interactions or functional redundancy between the 2 loci (Fig. 4e; Figure S8b, c).

Taken together, these results confirm a role for *GEMIN2* in the modulation of plant growth in response to the temperature and extend this function to a broader range of ambient temperatures. Although GEMIN2 is not essential for plant viability, it represents an important regulator of temperature-dependent plant development.

### Arabidopsis GEMIN2 differs from its human and yeast counterparts

The absence of an obvious ortholog of SMN, as well as most other GEMIN proteins, strongly suggests that plants employ an assembly mechanism distinct from that of metazoans.

To test whether GEMIN2, the only component of the SMN complex that we could confidently identify in Arabidopsis, has diverged functionally across taxa, we expressed codon-optimized CDSs of the human GEMIN2 ortholog (*HsGEMIN2*) and the yeast GEMIN2 ortholog (*ScBRR1P*) in the Arabidopsis *gemin2-2* mutant. Orthologs from these 2 species were selected because humans possess a canonical multiprotein SMN complex composed of SMN, GEMIN2, and several additional GEMIN proteins, whereas *S. cerevisiae* contains only the GEMIN2 ortholog BRR1P (Kroiss et al. 2008). We found that neither human nor yeast *GEMIN2* was able to rescue the developmental defects of the *gemin2-2* plants grown at 16 °C when expressed under the *AtGEMIN2* promoter (Fig. 4f). In total, we analyzed more than 30 independent primary transformants (T1) per construct, and all displayed the characteristic *gemin2-2* mutant phenotype (Fig. 4f). In contrast, expression of the Arabidopsis GEMIN2 CDS from the same promoter partially complemented the mutant phenotype (Fig. 4d).

**Figure 5:**
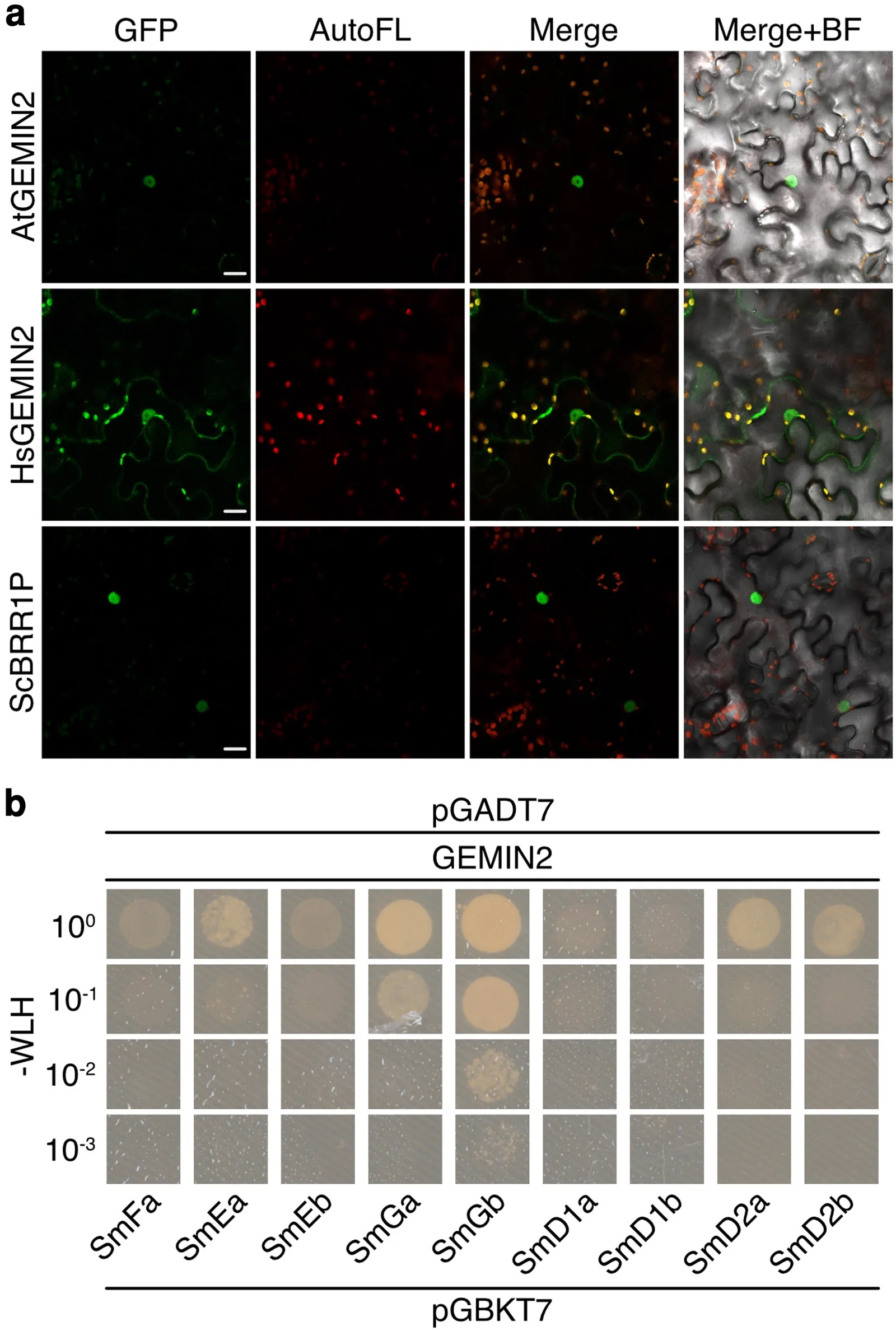
Plant GEMIN2 differs from the human ortholog. a), Subcellular localization of GFP-GEMIN from human (*pGEMIN2::GFP-HsGEMIN2_CDS*), yeast (*pGEMIN2::GFP-BRR1P_CDS*) and Arabidopsis (*pGEMIN2::GFP-AtGEMIN2_CDS*) in tobacco leaves. Scale bars: 20 *μ*m. b), Yeast-2-hybrid testing interaction between GEMIN2 and Sm proteins. pGADT7 and pGBKT7 vectors contribute AD and BD domains, respectively. Photos were taken after 4 d of growth on selective drop-out medium.

To support this negative result, we verified the expression of the transgenes and compared the subcellular localization of the human and yeast proteins with that of Arabidopsis *GEMIN2*. Importantly, transient expression of reporter proteins in which AtGEMIN2, HsGEMIN2, and ScBRR1P had been fused to GFP in tobacco leaves revealed that all 3 proteins accumulate predominantly in the nucleus (Fig. 5a), with GFP-tagged HsGEMIN2 also detectable at the cell periphery. These observations suggest that functional divergence among GEMIN2 orthologs might contribute to the inability of human and yeast proteins to rescue the *gemin2-2* mutant, beyond potential differences in expression or subcellular localization. To further characterize the role of GEMIN2 in plant snRNP assembly, we tested its ability to interact with those Sm proteins known to associate with GEMIN2 in metazoans, ie SmE, SmF, SmG, SmD1, and SmD2. Y2H analyses revealed that Arabidopsis GEMIN2 behaves differently from its human counterpart, interacting strongly only with SmGa and SmGb and, to a lesser extent, with SmD2a, SmD2b, and SmEa (Fig. 5b; Figure S9).

Together, these results indicate that plant GEMIN2 differs in its function from its human and yeast counterparts. Although the evolutionary processes underlying these differences remain unclear, our data suggest that GEMIN2 proteins have undergone substantial functional diversification across eukaryotic lineages. Consistent with this view, plants may not require a canonical SMN complex to support snRNP assembly.

## Discussion

RNA splicing is of fundamental importance in plant–environment interactions, representing one of the earliest steps through which plants adjust their transcriptome and proteome in response to external stimuli. In metazoans, the step-wise assembly of snRNPs, which form the catalytic core of the spliceosome, is facilitated by 2 protein complexes, the methylosome and the SMN complex. Because the core components of these complexes are evolutionary conserved, snRNP assembly has generally been assumed to follow similar principles across eukaryotes, including plants (Matera and Wang 2014; Dikaya et al. 2021). However, accumulating evidence suggests that important differences in snRNP assembly exist between plants and metazoans. Several components of the methylosome and SMN complex, including PICLN, PRMT5, and GEMIN2 have been implicated in the regulation of plant growth and environmental responses (Pei et al. 2007; Zhang et al. 2011; Fu et al. 2013; Schlaen et al. 2015; Hu et al. 2017; Mateos et al. 2023). Here, we investigated the contribution of these genes to Arabidopsis development across the physiologically relevant ambient temperature range from 16 to 27 °C, under which all mutants analyzed remain viable.

We found that the phenotype of the *gemin2* mutant, which was previously reported to be seedling lethal at 4 °C (Schlaen et al. 2015), is actually strongly temperature-dependent. At 16 °C, *gemin2* plants displayed strong developmental defects but remained viable, and these defects progressively diminished with increasing temperatures. In parallel, we investigated the composition of the SMN complex in Arabidopsis. We were unable to detect a direct interaction between GEMIN2 and AtSPF30, which has previously been proposed to function as a plant SMN, and our Y2H screens did not identify proteins with features characteristic of canonical metazoan SMN complex components. Although these observations are based largely on negative evidence, they nevertheless suggest that Arabidopsis lacks a canonical SMN complex and that GEMIN2 may represent its only clearly conserved component.

While substantial divergence in such a fundamental cellular process such as snRNP assembly may appear unexpected, similar evolutionary reductions of the SMN complex have been reported in fungi. For example, the complex is reduced to 5 proteins in *S. pombe* and a single GEMIN2 ortholog, BRR1P, in *S. cerevisiae* (Kroiss et al. 2008; Hu et al. 2023). Nevertheless, the inability of either human GEMIN2 or yeast BRR1P to complement the Arabidopsis *gemin2* mutant indicates that these proteins have functionally diverged during evolution. Consistent with this interpretation, Arabidopsis GEMIN2 interacted in Y2H assays only with a subset of the Sm proteins (SmGa, SmGb, SmEa, SmD2a, SmD2b) predicted based on its human counterpart. Importantly, these protein–protein interactions nevertheless establish direct connection between GEMIN2 and the Sm proteins in plants and are therefore consistent with a role in snRNP assembly. Our conclusions are further supported by recent bioinformatics analyses, suggesting that proteins containing characteristic SMN features are present in some plant lineages but absent from Arabidopsis (Gupta et al. 2021). Notably, candidate proteins identified in legumes, cotton, corn, and jute possess motifs related to the glycine zipper motif of the C-terminal YG box, a highly conserved domain required for SMN function in metazoans (Wirth 2000; Gupta et al. 2021). The apparent absence of such proteins in Arabidopsis further supports the idea that this species may employ a modified snRNP-assembly pathway. Taken together, our findings suggest that Arabidopsis lacks a canonical SMN complex and that GEMIN2 may represent its only conserved component. More broadly, our results indicate that, despite conservation of several key factors, the mechanisms controlling snRNP biogenesis in Arabidopsis differ substantially from those in metazoans.

Additional support for divergence of the plant snRNP assembly pathway comes from our analyses of the plant homologs of the methylosome components PICLN and PRMT5. In metazoan, SDM of specific Sm proteins is considered an important step in snRNP assembly. To investigate the contribution of this process in Arabidopsis, we conducted a detailed phenotypic analysis of the *prmt5*, *picln*, and *smd1b* mutants across different temperatures. *SmD1b* was selected because it encodes a predicted methylosome target and a knockout mutant of the gene has been described previously (Elvira-Matelot et al. 2016). Similar to *gemin2-2*, the pleiotropic phenotypes of *picln* and *smd1b* mutants observed at 23 °C became even more pronounced at lower ambient temperature but were largely alleviated at 27 °C. In contrast, the phenotype of *prmt5-1* remained relatively stable across the entire temperature range examined. These observations suggest that although all 3 genes have been linked to snRNP biogenesis, their contributions to plant development and temperature responses are not equivalent.

The contrasting phenotypes of *picln* and *prmt5* mutants, together with the limited temperature sensitivity of *prmt5-1*, prompted us to consider 2 possible explanations. First, Arabidopsis could possess additional methyltransferases that act redundantly with PRMT5. Alternatively, SDM of Sm proteins might play a less important role in snRNP assembly in plants than it does in metazoans. The first alternative seems unlikely based on our Y2H analyses, which did not detect interactions between PRMT7, PICLN, and any of the tested Sm proteins. Although these data do not exclude functional redundancy, they provide no evidence for a second methyltransferase operating through the same molecular framework as PRMT5. We therefore decided to test the contribution of SDM of Sm proteins to snRNP assembly using a combination of genetic, complementation, and protein–protein interaction approaches.

Our findings indicate that PRMT5 function is dispensable for plant viability but required for proper plant development under the conditions tested. These findings are consistent with the possibility that SDM is not absolutely required for SmD1b function and, consequently, snRNP assembly in Arabidopsis. However, previous studies have reported a direct interaction between PRMT5 and PICLN and lethality of the double mutant (Huang et al. 2016; Mateos et al. 2023), suggesting that the relationship between these proteins may be more complex than anticipated. In this regard, it is noteworthy that PRMT5 and PICLN participate in the DNA damage response in human cells independently of MEP50 and that PRMT5 has an independent role in splice-site selection as key regulator of alternative splicing fidelity (Owens et al. 2020; Beckel et al. 2025). As MEP50 does not appear to be conserved in plants, it seems possible that PRMT5 and PICLN also perform functions outside of snRNP assembly in plants. Although this possibility remains speculative, it may help explain some of the genetic interactions reported previously.

Additional support for partially independent functions of PICLN and PRMT5 comes from RNA-seq analyses performed by Mateos et al. (2023). These authors reported both overlapping and distinct molecular functions for PICLN and PRMT5 and showed that the splicing profile in *prmt5* plants differed substantially from those of *picln* and *gemin2* mutants, which were more similar to each other. Interestingly, despite these molecular differences, both *picln* and *prmt5* were reported to exhibit late flowering in that study. This contrasts with our finding that *picln* flowers early under our experimental conditions. One possible explanation for this discrepancy is the difference in growth conditions, as Mateos et al. (2023) grew plants under a 12 h light/12 h dark photoperiod, whereas our experiments were conducted under long-day photoperiod (16 h light/8 h night).

Given these apparently conflicting results, we sought to test more directly whether the PRMT5–PICLN methylosome is essential for plant development. By growing plants at 27 °C, a condition under which the developmental defects of *picln-2* are largely suppressed, we were able to establish the *picln-2 prmt5-1* double mutant, previously reported to be lethal. Once established, the double mutant remained viable at both 16 and 23 °C, although its phenotype was more severe than that of either single mutant. Germination efficiency and seedling development were particularly compromised. These observations indicate that while PRMT5 and PICLN make important contributions to plant growth and development, neither appears to be strictly required for viability under the conditions examined.

Taken together, our genetic, molecular, and phenotypic data suggest that the contribution of PICLN–PRMT5 to snRNP assembly in Arabidopsis differs from that described in metazoans, where the importance of individual components often has severe or lethal consequences. Furthermore, the apparent tolerance of Arabidopsis to reduced Sm-protein methylation is consistent with the absence of a canonical SMN protein, whose TUDOR domain preferentially recognizes symmetrically dimethylated Sm proteins in metazoans. In agreement with this interpretation, the ability of *gSmD1b^Lys^* to complement the *smd1b* mutant indicates that the C-terminal region containing methylated arginine residues is not strictly required for biological function under the conditions tested.

Overall, our results support a model in which snRNP assembly in Arabidopsis relies less heavily on the canonical methylosome–SMN pathway than in metazoans. Although dedicated assembly factors such as PICLN and GEMIN2 clearly contribute to efficient plant development, their functions may be more auxiliary than essential. These findings raise the possibility that plants, similar to *S. cerevisiae*, employ a modified assembly pathway that relies to a greater extent on the intrinsic capacity of Sm proteins to assemble into functional complexes, possibly assisted by other still unknown accessory factors.

Finally, an increasing number of splicing-related mutants have recently been reported to exhibit temperature-sensitive phenotypes. Most of these mutants display enhanced defects at low temperature, although a warm-sensitive *lsm7* knock-down mutant has recently been reported, highlighting the importance of RNA processing across the entire physiological temperature range (Nardeli et al. 2023). The reasons why mutations affecting components of related molecular pathways produce such distinct temperature-response phenotypes remain poorly understood. Future studies will be required to disentangle the role of RNA splicing in temperature acclimation and whether manipulation of these pathways can be exploited to enhance plant resilience to environmental fluctuations.

## Materials and methods

### Plant material, growth conditions, and ambient temperature phenotyping

All genotypes used in this research were in Columbia background and mutants were obtained from Nottingham Arabidopsis Stock Center (NASC) or provided by colleagues: *gemin2-1* (SALK_142993), *gemin2*-2 (SAIL_567_D05), *prmt5-1* (SALK_065814), *prmt5-2* (SALK_095085), *picln-1* (GABI675_D10), *picln-2* (SALK_050231), *gemin2-like-1* (SAIL_691_C12), *atspf30-1* (SALK_052016), *atspf30-2* (SALK_081292), *prmt7-1* (SALK_039529), *smd1b-1* (*sgs14* mutant, from Elvira-Matelot et al. 2016). All mutants were genotyped by PCR using the primers listed in Table S4. Seeds were stratified at 4 °C in the dark for 72 h before being sown on soil. Plants were grown under long-day conditions (LD), 16 h light/8 h dark, in Percival chambers equipped with full range white LED illumination at an intensity of 120 to 150 *µ*mol*m^−2^s^−1^, with controlled humidity (RH 70%) at different constant ambient temperatures (16, 23, and 27 °C). Flowering time was measured as the number of leaves originating from the main shoot meristem and the number of days until the floral buds appeared; rosette diameter was measured after bolting once the stem was 1 cm long. A 2-way ANOVA was performed to assess the statistical significance of the interaction between the 4 genotypes (Col-0, *gemin2-2*, *picln-2*, and *prmt5-1*) and temperatures (16, 23, and 27 °C). Germination and survival rates of Col-0, *prmt5-1*, *picln-2* and the *prmt5-1 picln-2* double mutant were estimated by sowing seeds on half-strength MS agar plates without sugar. Three independent plates were used per genotype at each temperature. The total number of screened seeds for Col-0, *picln-2*, *prmt5-1*, and *prmt5-1 picln-2* at each temperature is as follows: 16 °C: 146, 178, 144, 162; 23 °C: 129, 153, 156, 146; 27 °C: 132, 114, 120, 194.

### DNA vectors and plant transformation

With the exclusion of the sequences listed in Table S5 that were synthesized by Eurofins, the sequences were amplified by PCR from cDNA or genomic DNA and cloned into GreenGate entry vectors by using the GreenGate system (Lampropoulos et al. 2013). Final constructs were assembled in the pGREENII-based GreenGate plant binary destination vector pGGZ003 and transformed into *Agrobacterium tumefaciens* (strain GV3101 pMP90 pSoup) by electroporation (Gene Pulser Xcell system). Arabidopsis plants were transformed by the floral dip method. BASTA selection (0.1%, v/v) was used for screening the transgenic lines on soil. Lists of the PCR primers used for cloning and the vectors generated in this study can be found in Tables S4 and S6.

### Phylogenetic analysis

Protein sequence similarity searches were performed using the BLASTP and DELTA-BLAST algorithms implemented at the NCBI BLAST database. Query protein sequences were retrieved from UniProt and used to search against the non-redundant (nr) protein database at the NCBI. BLASTP searches were conducted using default parameters, with an expectation value (*E*-value) threshold of 1e-5 for significant hits. DELTA-BLAST searches were performed to improve detection of distant homologs by incorporating conserved domain information from the conserved domain database. GEMINs and SMN phylogenetic trees were generated with PhylomeDB v5 (Fuentes et al. 2022). The *Homo sapiens* reference phylome was queried to retrieve the pre-computed maximum-likelihood phylogenetic trees and multi-sequence alignments associated with each seed sequence.

### Y2H assay and library screening

For testing protein–protein interactions in a one-to-one Y2H assay, the CDSs of the tested genes were cloned into the yeast vectors pGADT7 or pGBKT7 adapted to the GreenGate cloning system (Zacharaki et al. 2022). Oligos used in the cloning are listed in Table S4. Pairs of vectors, including negative controls, were used to co-transform yeast strain AH109 and colonies carrying both vectors were selected on SD medium without tryptophan (-W) and leucine (-L) (TaKaRa 630317) at 28 °C. After 6 d, protein–protein interactions were tested by growing serial dilutions on SD drop-out medium lacking tryptophan (-W), leucine (-L), and histidine (-H) (TaKaRa 630319).

For the Y2H Library Screening, a cDNA expression library was generated from mRNA pooled from the aerial part of Col-0 seedlings grown at 16 °C, harvested 11 and 13 d after sowing, 3, 7, and 10 h after daylight started. The mRNA was used to create a Gateway-compatible cDNA entry library by using the CloneMiner cDNA library Construction Kit (Invitrogen) according to the manufacturer's instructions. The final cDNA library in the pDEST22 vector had a titer of 4.9 × 10^6^ cfu·mL^−1^. The *GEMIN2* CDS was cloned into the bait pDEST32 vector. The cDNA library screening was performed as described by de Folter and Immink (2011).

### Reverse transcription quantitative PCR

Total RNA was extracted using the Qiagen Plant RNeasy kit and treated with RNase-free DNaseI (Thermo Scientific) to remove DNA contamination. Complementary DNA (cDNA) was synthesized using the RevertAid First Strand cDNA Synthesis kit (Thermo Fisher) in accordance with the manufacturer's instructions. RT-PCR was carried out using a CFX96 Real-time System (Biorad). and SYBR Green Master Mix (Bioline). The relative expressions were calculated using the 2^(−ΔΔCT)^ method. For each sample, 3 biological and 3 technical replicates were used. Primers used in reverse transcription quantitative PCR (RT–qPCR) are listed in Table S4.

### Transient expression of fluorescent fusion proteins in tobacco leaves

Three- to five- week-old *Nicotiana benthamiana* plants grown under long-day (16 h light/8 h darkness) conditions were used for this experiment. *Agrobacterium tumefaciens* cultures expressing tested constructs were inoculated and grown to saturation in liquid super optimal broth (SOC) with catabolite suppression media supplemented with appropriate antibiotic combinations. A 100 *μ*L inoculum from each culture was transferred to separate 15 or 50 mL conical centrifuge tubes containing fresh antibiotic-supplemented SOC media and incubated overnight. The overnight cultures were subsequently centrifuged, supernatant media discarded, and bacteria resuspended in infiltration solution (10 mm MgCl_2_, 10 mm 2-(N-morpholino) ethanesulfonic acid, 200 *µ*m acetosyringone, pH adjusted with KOH to 5.6). The volume of infiltration solution used for resuspension was equal to the volume of liquid media used for the overnight incubation. The resuspended cultures were then diluted in infiltration solution to an OD_600_ between 0.5 and 1. The resuspended cultures were incubated with gentle shaking for 3 h at room temperature. For transient expression of *pGEMIN2::GFP-HsGEMIN2_CDS*, *pGEMIN2::GFP-BRR1P_CDS* and *pGEMIN2::GFP-AtGEMIN2_CDS*, 5 mL of culture expressing each construct was mixed with 5 mL of culture expressing the RNA silencing suppressor p19 in 15 mL conical centrifuge tubes. These 10 mL mixtures were then infiltrated using a syringe into young, spongy *N. benthamiana* leaves. Each construct was infiltrated into at least 2 leaves, and different *N. benthamiana* individuals were used for each biological replicate. Leaf fragments from infiltrated regions were imaged using a ZEISS LSM800 (2 biological replicates) or LSM880 (1 biological replicate) confocal laser scanning microscope. GFP was excited using a 488 nm laser.

### BiFC analysis

For BiFC analysis, *A. tumefaciens* lines carrying constructs expressing C-citrine and N-citrine fragments were inoculated separately. The protocol used was identical to the transient expression protocol described in the preceding section until the 3 h incubation of diluted overnight cultures resuspended in infiltration solution. After the 3 h incubation, 5 mL each of C- and N-citrine cultures were mixed in a 15 mL conical centrifuge tube with 1 mL of the RNA silencing suppressor p19. *Nicotiana benthamiana* leaves were infiltrated as described in the previous section. Citrine was excited using a 513 nm laser when using the LSM800 (2 biological replicates) or a 488 nm laser for the final biological replicate. A minimum of two 1.5 × 1.5 cm regions within the infiltrated area were screened per infiltrated leaf.

## Supplementary Material

kiag667_Supplementary_Data

## Data Availability

The data underlying this article are available in the article and in its online supplementary files.
