# Supplementary material for: Methylosome and survival motor neuron complexes are dispensable for plant viability in Arabidopsis thaliana
Source: Plant Physiol. 2026 Sep 5;202(1):kiag667. doi: 10.1093/plphys/kiag667 (PMC13596061; doi:10.1093/plphys/kiag667)
Supplement: Supplementary file 1 [file kiag667_supplementary_data.zip › Goretti_et_al_snRNP-assembly_Supplemental_Figures_R1.docx]

**Supplemental Figure S1. Phenotypes of single and double methylosome mutants.** A**,** RT-qPCR analysis of *PICLN* and *PRMT5* in Col-0 and *picln-2* or *prmt5-1*, respectively from plants grown at 23°C, normalized against *TUB2*. Error bars represent standard error. B, C, Flowering time of Col-0 and mutant genotypes at three different ambient temperatures. D, Phenotype of Col-0, *picln-2*, and *prmt5-1* grown at 23°C (top) and 27°C (bottom) for 18 and 25 days-after-sowing, respectively. Scale bar: 1 cm. Col-0 plants are the same shown in Supplemental Figure S3. E, Phenotype of Col-0, *picln-2,* and *picln-1* grown at 16°C for 35 days-after-sowing. F, RT-qPCR analysis of *GEMIN2*, *PICLN*, and *PRMT5* in Col-0 grown at three different ambient temperatures, normalized against *TUB2*. Error bars represent standard error. G, Flowering time of Col-0 and *prmt5-1 picln-2* double mutant genotypes at three different ambient temperatures. Numbers on the x-axis in B, C, and G indicate the number of individuals per genotype and condition. One-way ANOVA was used for statistical analysis in B,C, and G. Different letters indicate categories that are statistically different after Tukey-Kramer correction (p≤0.05). DAS, Days After Sowing.

**Supplemental Figure S2. PRMT7 does not interact with PICLN or Sm proteins.** A, Y2H interaction assay between PRMT7 and PICLN or Sm proteins. Dilution series of yeast cells on control (SD-WL; left) and selective medium (SD-HWL; right). Photos were taken four days after plating. pGADT7 and pGBKT7 contribute the AD and BD domains, respectively. B, RT-qPCR analysis of *PRMT7* in Col-0 and *prmt7-1* from plants grown at 23°C, normalized against *TUB2*. Error bars represent standard error. C, Flowering time of Col-0 and *prmt7-1* at three different temperatures. Numbers on the x-axis indicate the number of individuals per genotype and condition. One-way ANOVA was used for statistical analysis. Different letters indicate categories that are statistically different after Tukey-Kramer correction (p≤0.05). DAS, Days After Sowing.

**Supplemental Figure S3. SDM is not essential for SmD1b function.** A, Alignment of SmD1b wild-type protein and mutated forms. The green box indicates the mutagenized C-term region. The blue box highlights arginine at position 61, part of the not mutagenized RG motif. B, Dilution series of yeast cells grown for four days on control medium (SD-WL) for testing interaction between three different SmD1 variants and two SmD2 proteins and PRMT5. pGADT7 and pGBKT7 contribute the AD and BD domains, respectively. Corresponding growth of cells on drop-out medium (SD-WLH) is shown in Figure 2A. C, Phenotypes of Col-0 and *smd1b-1* plants grown at 23°C (top) and 27°C (bottom) for 25 and 18 DAS, respectively. Col-0 plants are the same shown in Supplemental Figure S1. D, Flowering time of Col-0 and *smd1b-1* in days-after-sowing at three different ambient temperatures. E, F, Flowering time of different T1 lines compared to wildtype and *smd1-b* mutant grown at 16°C. Numbers on the x-axis in D-F indicate the number of individuals per genotype and condition. One-way ANOVA was used for statistical analysis. Different letters indicate categories that are statistically different after Tukey-Kramer correction (p≤0.05). DAS, Days After Sowing.

**Supplemental Figure S4. Phenotype of T1 plants expressing wild-type and mutated *SmD1b*.** A, Phenotype of *smd1b-1* transformed with gSmDb1^Lys^. Plants show the *smd1b-1* mutant phenotype with irregular leaves margins and flat leaves. B, Picture of T1 plants transformed with the wild-type gSmDb1 (top) and gSmDb1^Lys^ constructs. Arrows indicate plants with *smd1b-1-like* leaf phenotype.

**Supplemental Figure S5. *AtSPF30* regulates flowering time.** A, Flowering time of Col-0 and two Arabidopsis *spf30* alleles at three different ambient temperatures. Numbers on the x-axis indicate the number of individuals. One-way ANOVA was used for statistical analysis. Different letters indicate categories that are statistically different after Tukey-Kramer correction (p≤0.05). DAS, Days After Sowing. B, Dilution series of yeast transformed with GEMIN2, AtSPF30, ASIL1 and SmGb constructs on control media (SD -WL). Photos were taken after four days after plating. pGADT7 and pGBKT7 contribute the AD and BD domains, respectively. C, Dilution series of yeast transformed with GEMIN2 and SmGb, or ASIL1 and SPF30 (positive controls) constructs on selective media (SD -WLH). Negative controls were were also added. Photos were taken after four days after plating. pGADT7 and pGBKT7 contribute the AD and BD domains, respectively. D, Three bioreps of BiFC analysis in tobacco leaves testing the interaction N-citrine:CARP9 + SPF30:C-citrine, used as negative control. The BiFC signal was detected 48 hours after infiltration by confocal microscopy. Scale bars represent 20 µm.

**Supplemental Figure S6. SMN1 ortholog in plants.** A, Phylogenetic tree of SMN1 obtained using PhylomeDB via UniProt. Branch lengths are proportional to evolutionary divergence, and the scale bar indicates the number of substitutions per site. Q16637 correspond to the UniProt identification code for human SMN1. No candidates were identified among plants. B, Result of the domain analysis (DELTA-BLAST) showing the homology region between human SMN1 and Arabidopsis TSN1, corresponding to the Tudor domain.

**Supplemental Figure S7. Phylogenetic analysis of GEMIN proteins across different clades.** Phylogenetic trees of GEMIN2–GEMIN7 protein families were obtained using PhylomeDB via UniProt. The trees depict orthologous relationships across multiple species. Branch lengths are proportional to evolutionary divergence, and the scale bar indicates the number of substitutions per site. Gene identifier for Arabidopsis thaliana is indicated where applicable.

**Supplemental Figure S8. Phenotypes of *gemin2* and *gemin2 gemin2-like-1*.** A, Phenotype of Col-0 and *gemin2-1* grown at 16°C at 35 days-after-sowing. Scale bar: 1 cm. B,C, Flowering time of Col-0, *gemin2-2*, *gemin2-like-1*, and *gemin2-2 gemin2-like-1* at three different ambient temperatures. Numbers on the x-axis indicate the number of individuals. One-way ANOVA was used for statistical analysis. Different letters indicate categories that are statistically different after Tukey-Kramer correction (p≤0.05). DAS, Days After Sowing. D, Gene structure at the *GEMIN2-like* locus. Dark-grey box indicates 5’UTR, light-grey boxes are exons, white boxes are introns. The triangle indicates the T-DNA insertion point. E, RT-qPCR analysis of *GEMIN2-LIKE* in Col-0 and *gemin2-like-1* mutant grown at 23°C, normalized against *TUB2*. Error bars represent standard error.

**Supplemental Figure S9. Y2H controls for GEMIN2 and Sm interaction.** Dilution series of yeast transformed with GEMIN2 and Sm constructs on control medium (SD-WL). Photos were taken after four days after plating. pGADT7 and pGBKT7 contribute the AD and BD domains, respectively.
