# Supplementary material for: Methylosome and survival motor neuron complexes are dispensable for plant viability in Arabidopsis thaliana
Source: Plant Physiol. 2026 Sep 5;202(1):kiag667. doi: 10.1093/plphys/kiag667 (PMC13596061; doi:10.1093/plphys/kiag667)
Supplement: Supplementary file 1 [file kiag667_supplementary_data.zip › Goretti_et_al_snRNP-assembly_Supplemental_Tables_R1.docx]

| **Supplemental Table S1. Rosette diameter (cm) of wild-type and main mutants described in this study grown at 16°C, 23°C, and 27°C.*** | | | |
| --- | --- | --- | --- |
| **Genotype** | **16°C** | **23°C** | **27°C** |
| Col-0 | 8.35 ± 0.53 | 8.83 ± 0.56 | 6.59 ± 0.44 |
| *gemin2-2* | 0.88 ± 0.38 | 4.09 ± 0.43 | 6.29 ± 0.72 |
| *picln-2* | 3.38 ± 0.53 | 7.00 ± 0.71 | 5.88 ± 0.35 |
| *prmt5-1* | 7.60 ± 0.30 | 10.17 ± 0.61 | 8.73 ± 0.61 |
| *prmt5-2* | 7.10 ± 0.58 | 9.85 ± 0.46 | 9.01 ± 0.31 |
| *picln-2 prmt5-1* | 7.00 ± 0.33 | 8.89 ± 0.55 | 7.15 ± 0.82 |
| *smd1b-1* | 4.53 ± 0.56 | 6.32 ± 0.88 | 5.32 ± 0.43 |

* taken when stem was 1 cm high

**Supplemental Table S3. List of GEMIN2 interactors identified in Y2H screening.**

| **Locus** | **Gene Model Description** | **Gene Type** | **Primary Gene Symbol** |
| --- | --- | --- | --- |
| AT3G60300 | RWD domain-containing protein;(source:Araport11) | protein_coding |  |
| AT3G47070 | thylakoid soluble phosphoprotein;(source:Araport11) | protein_coding |  |
| AT1G29880 | glycyl-tRNA synthetase / glycine-tRNA ligase;(source:Araport11) | protein_coding |  |
| AT3G06150 | cytochrome P450 family protein;(source:Araport11) | protein_coding |  |
| AT2G23120 | Late embryogenesis abundant protein, group 6;(source:Araport11) | protein_coding |  |
| AT3G27160 | GHS1 encodes plastid ribosomal protein S21The mRNA is cell-to-cell mobile. Required for photosynthesis and C/N balance. | protein_coding | (RPS21) |
| AT1G62380 | Encodes a protein similar to 1-aminocyclopropane-1-carboxylic oxidase (ACC oxidase). Expression of the AtACO2 transcripts is affected by ethylene. | protein_coding | ACC OXIDASE 2 (ACO2) |
| AT4G34610 | Acts as a negative regulator of xylem vessel cells as a protein complex together with OVATE FAMILY PROTEIN1 (OFP1), OFP4, and MYB75. | protein_coding | BEL1-LIKE HOMEODOMAIN 6 (BLH6) |
| AT5G14740 | Encodes a beta carbonic anhydrase likely to be localized in the cytoplasm. Expression of its mRNA is seen in etiolated seedlings and points to a possible nonphotosynthetic role for this isoform. | protein_coding | CARBONIC ANHYDRASE 2 (CA2) |
| AT4G34100 | Encodes a putative E3 ubiquitin ligase that is involved in cuticular wax biosynthesis and regulates 3-hydroxy-3-methylglutaryl-CoA reductase (HMGR) activity. HMGR catalyzes the major rate-limiting step of the mevalonic acid (MVA) pathway from which sterols and other isoprenoids are synthesized. Lines carrying a recessive mutation in this locus have reduced chain-length distribution, weakly glaucous stem surface, and has reduced fertility in early flowers, non-spreading floret, downward cupped leaves, leaf waxes nearly pure C24 and C26 acid. | protein_coding | ECERIFERUM 9 (CER9) |
| AT4G23250 | cysteine-rich receptor-like protein kinase 17;(source:Araport11) | protein_coding | EMBRYO DEFECTIVE 1290 (emb1290) |
| AT5G45680 | Peptidyl-Prolyl Isomerase located in chloroplast thylakoid lumen The mRNA is cell-to-cell mobile. | protein_coding | FK506-BINDING PROTEIN 13 (FKBP13) |
| AT5G62270 | Ribosomal protein L20;(source:Araport11). Required for proper mitochondrial cristae formation. Expressed throughout plant. Mutants are defective in late stages of megagametogenesis. Pollen tube defective. Gametophytic lethality is probably due to mitochondrial disfunction. | protein_coding | GAMETE CELL DEFECTIVE 1 (GCD1) |
| AT4G08350 | global transcription factor group A2;(source:Araport11) | protein_coding | GLOBAL TRANSCRIPTION FACTOR GROUP A2 (GTA2) |
| AT2G05520 | Encodes a glycine-rich protein that is expressed mainly in stems and leaves. AtGRP3 functions in root size determination during development and in Al stress. mRNA levels are upregulated in response to ABA, salicylic acid and ethylene but downregulated in response to desiccation. The mRNA is cell-to-cell mobile. | protein_coding | GLYCINE-RICH PROTEIN 3 (GRP-3) |
| AT4G13550 | Heat stress inducible plastid monogalactosyldiacylglycerol lipase. | protein_coding | HEAT INDUCIBLE LIPASE1 (HIL1) |
| AT1G79920 | Heat shock protein 70 (Hsp 70) family protein;(source:Araport11) | protein_coding | HEAT SHOCK PROTEIN 70-15 (Hsp70-15) |
| AT5G51570 | SPFH/Band 7/PHB domain-containing membrane-associated protein family;(source:Araport11) | protein_coding | HYPERSENSITIVE INDUCED REACTION 3 (HIR3) |
| AT1G30380 | Encodes subunit K of photosystem I reaction center. The mRNA is cell-to-cell mobile. | protein_coding | PHOTOSYSTEM I SUBUNIT K (PSAK) |
| AT2G30570 | Encodes PsbW, a protein similar to photosystem II reaction center subunit W. Loss of PsbW destabilizes the supramolecular organization of PSII. | protein_coding | PHOTOSYSTEM II REACTION CENTER W (PSBW) |
| AT1G06680 | Encodes a 23 kD extrinsic protein that is part of photosystem II and participates in the regulation of oxygen evolution. Phosphorylation of this protein is dependent on calcium. In plsp1-1 mutant plastids, the nonmature form of the protein localizes in the stroma. The mRNA is cell-to-cell mobile. | protein_coding | PHOTOSYSTEM II SUBUNIT P-1 (PSBP-1) |
| AT2G43460 | Ribosomal L38e protein family;(source:Araport11) | protein_coding | RIBOSOMAL PROTEIN EL38Z (EL38Z) |
| AT3G27830 | 50S ribosomal protein L12-A The mRNA is cell-to-cell mobile. | protein_coding | RIBOSOMAL PROTEIN L12-A (RPL12-A) |
| AT5G38420 | Encodes a member of the Rubisco small subunit (RBCS) multigene family: RBCS1A (At1g67090), RBCS1B (At5g38430), RBCS2B (At5g38420), and RBCS3B (At5g38410). Activated by OXS2 under the treatment of salt. | protein_coding | RUBISCO SMALL SUBUNIT 2B (RBCS2B) |
| AT5G38410 | Encodes a member of the Rubisco small subunit (RBCS) multigene family: RBCS1A (At1g67090), RBCS1B (At5g38430), RBCS2B (At5g38420), and RBCS3B (At5g38410). Functions to yield sufficient Rubisco content for leaf photosynthetic capacity. | protein_coding | RUBISCO SMALL SUBUNIT 3B (RBCS3B) |
| AT2G47110 | polyubiquitin gene The mRNA is cell-to-cell mobile. | protein_coding | UBIQUITIN 6 (UBQ6) |

**Supplemental Table S4. List of oligos used in this study.**

| **Target** | **Foward Sequence (5' -> 3')** | **Reverse Sequence (5' -> 3')** |
| --- | --- | --- |
| **Genotyping** | | |
| SALK_142993 (*gemin2-1*) | GTCTGTCTGTTTTCCTGCTCG | CTCATCCTCCTCTGTGTGGAG |
| SAIL_567_D05 (*gemin2-2*) | TCGAGATTACCATTGCCACTC | GTGGAATTAGCAGATATGCGG |
| SAIL_691_C12 (*gemin2-like-1*) | ATTTGGAATTCGCTTAGCCTC | GCTGGTGACTGAGTACGAAGG |
| SALK_052016 (*spf30-1*) | CGGAGTTAGTGCTGAAGCAAC | TTCCAGTCACACCCACTTTTC |
| SALK_081292 (*spf30-2*) | AAAACGGCAGAGATTTTACCG | CAGCAAAGCTCAAAATCGATC |
| GABI_675D10 (*picln-1*) | TTCATCTCCATTGGAATGACC | TACTGGATGAATCTAACGGCG |
| SALK_050231 (*picln-2*) | TGGCTAAAGGATACGCAGTTG | TGAACCATCTCTTCAGCATCC |
| SALK_065814 (*prmt5-1;skb1-1*) | TGGCCTTTTGAGATGAAAGAG | GCTCATAAAGATCTTGCGCAC |
| SALK_073624 (*prmt5-2; skb1-2*) | TGGTGGAAGGAAATGGTGTAG | CTCACAGACTCTCCCATCCAG |
| SALK_039529 (*prmt7-1*) | AGAGACTGGTGTGACCATTGG | GCTAGGAAACGAACGACACAG |
| *smd1b-1* | CTGCATCTGACTTGACCAGTG | TGTGTTGTATGCTTCGTCACC |
| **Cloning** | | |
| GEMIN2_promoter 1480bp (A-mod) | aacaggtctcaacctTATGCGGATCCGGTATAATC | aacaggtctcttgttGAGGCGGATGTGCGCAAAAC |
| GEMIN2_CDS (C-mod) | aacaggtctcaggctcaacaATGATGACAAACTCCGATTC | aacaggtctctctgaCTGTCCCATTTGGCCAAAG |
| SPF30_CDS (C-mod) | aacaggtctcaggctcaacaATGGTAGGAGGAGTAGAAG | aacaggtctctctgaTCATTCATCAGTGCCCTCAG |
| SmEa_CDS (C-mod) | aacaggtctcaggctcaacaATGGCGAGCACCAAAGTTCAAAG | aacaggtctctctgaCTTTCCCGTGTTCATCATCAGAG |
| SmEb_CDS (C-mod) | aacaggtctcaggctcaacaATGGCGAGCACCAAAGTTCAG | aacaggtctctctgaCTTGCCCGCGTTCATCATGAG |
| SmFa_CDS (C-mod) | aacaggtctcaggctcaacaATGGCGTTTGTATGTATGTGTG | aacaggtctctctgatcaTTAGTCTTGATCAGCGTCTTC |
| SmGa_CDS (C-mod) | aacaggtctcaggctcaacaATGAGTCGTTCAGGTCAGCCTCCG | aacaggtctctctgaCTAAGAAGATCTGCCCACTGGTTCA |
| SmD1a_CDS (C-mod) | aacaggtctcaggctcaacaATGAAGCTCGTCAGGTTTTTG | aacaggtctctctgaACGACCTCTGCCGCGACCAC |
| SmD1b_CDS (C-mod) | aacaggtctcaggctcaacaATGAAGCTCGTCAGGTTTTTG | aacaggtctctctgaACGACCACCACGGCCACGTCC |
| SmD2a_CDS (C-mod) | aacaggtctcaggctcaacaATGAGCAAGCCAATGGAAG | aacaggtctctctgaTCATTTTGGATTCCTGAGG |
| SmD2b_CDS (C-mod) | aacaggtctcaggctcaacaATGAGTAAACCAATGGAAG | aacaggtctctctgaTCACTTGGGGTTCCTGAGG |
| PRMT5_CDS (C-mod) | aacaggtctcaggctcaacaATGCCGCTCGGAGAGAGAG | aacaggtctctctgaCTAAAGGCCAACCCAGTACG |
| PICLN_CDS (C-mod) | aacaggtctcaggctcaacaATGGTGGCTGGTCTAAGAG | aacaggtctctctgaCTAGTGATCTTTGGTCTCAC |
| PRMT7_CDS (C-mod) | aacaggtctcaggctcaacaATGTCGCCTCTGTCTTCTC | aacaggtctctctgaAGAAATAGTATGAGTGACG |
| ASIL1 CDS (C-mod) | aacaggtctcaggctcaacaATGGAGGACGACGACGAG | aacaggtctctctgattaTCAGCTACTTACATTGC |
| SmD1bΔC_CDS (C-mod) | aacaggtctcaggctcaacaATGAAGCTCGTCAGGTTTTTG | aacaggtctctctgaCACTAACCTGCTGGAATCTTACCTGC |
| SmD1b^Lys^ _CDS (C-mod) | aacaggtctcaggctcaacaATGAAGCTCGTCAGGTTTTTG | aacaggtctctctgaTCATTAtttACCACCtttGCC |
| gSmD1b_genomic (C-mod) | aacaggtctcaggctcaacaATGAAGCTCGTCAGGTATGG | aacaggtctctctgaACGACCACCACGGCCACGTC |
| SmD1b_promoter 1578bp (A-mod) | aacaggtctcaacctTAGTTTCAGGTTAAAGTTGATTTG | aacaggtctcttgttGGTGAGAGAATGAAGAAGAAG |
| SmD1b_terminator (E-mod) | aacaggtctcactgcGCATTTTGCAGTAATCAAAAG | aacaggtctcttagtATTCATCACATGCTTAGCATAAG |
| **RT-qPCR** | | |
| GEMIN2 | ACGATGCAATAACTATGGAAG | TCTGAGCTTCAGATTCTTGTTC |
| PRMT5 | AACTTCTCAACGAAAGTCAAC | CCAGCTGAACATGTTTGGTG |
| PRMT7 | AAGCCATTGCTGATTCAAATG | TTCTGTCCTTCCAGAATCTGAG |
| PICLN | TCAAAGATCAGAGAGATGAGGC | CATCTGATCAGCACTAAACAC |
| GEMIN2-LIKE | TTGAAGAATCACAAGAGTATCC | TTGTAATCGTCATTATCTTCAG |
| SPF30 (in *spf30-1*) | GCGAGTGGTATGATGCGACC | TGGTAGCTTGAGCTAATCTCTC |
| SPF30 (in *spf30-2*) | AATGATTGGCAACAGTTTCAG | GGAAATCTGTCAAACCTTTC |
| TUB2 | GAGCCTTACAACGCTACTCTGTCTGTC | ACACCAGACATAGTAGCAGAAATCAAG |

**Supplemental Table S5. List of synthesized sequences (Eurofins).**

**HsGEMIN2_CDS (C-mod)**

aacaggtctcaggctcaacaATGAGAAGAGCTGAACTTGCGGGGTTGAAAACGATGGCATGGGTTCCTGCTGAATCTGCAGTGGAAGAGTTAATGCCTAGACTCTTGCCAGTAGAGCCATGTGATCTCACTGAGGGATTTGACCCTTCAGTTCCTCCTAGAACACCGCAAGAGTACCTACGTAGGGTGCAGATTGAAGCCGCTCAATGTCCTGACGTTGTTGTCGCTCAGATTGATCCCAAGAAGCTCAAGAGAAAACAGTCTGTGAATATCTCTCTGTCTGGATGTCAACCAGCTCCAGAGGGATATAGTCCGACCTTGCAATGGCAACAGCAACAGGTTGCTCAGTTCTCCACTGTTAGGCAGAATGTGAACAAACATCGATCACATTGGAAGAGCCAACAACTCGATTCAAATGTGACAATGCCGAAATCCGAAGATGAGGAAGGTTGGAAGAAGTTCTGTTTGGGCGAGAAACTGTGTGCAGATGGTGCAGTTGGTCCAGCAACGAACGAGAGTCCTGGGATAGACTATGTGCAAATAGGATTTCCACCCTTATTGAGCATCGTATCGAGAATGAACCAAGCCACTGTTACCTCTGTCCTCGAGTACCTTTCGAACTGGTTTGGTGAGCGTGATTTCACACCAGAACTAGGCAGATGGCTTTACGCGCTTCTAGCTTGCCTTGAAAAGCCTCTTCTCCCTGAAGCTCACAGTCTAATCAGGCAGTTGGCTAGGCGTTGCTCAGAGGTACGGTTGCTTGTCGATTCCAAAGACGATGAACGAGTCCCAGCCTTAAATCTTCTGATTTGCCTTGTTTCTCGGTATTTTGACCAAAGAGATTTAGCGGATGAACCGAGCtcagagagacctgtt

**ScBRR1P_CDS (C-mod)**

aacaggtctcaggctcaacaATGAAGAGAGGAGAAAGTCAAGCACCAGACGCAATCTTTGGCCAATCAAGAGCATTCGCTCTTAGTGACTCTTCGGTTAATCCTGATGTGATCGAGTACCTTAAAAGCGTTCGACAAGAAGCACTCAGGACCAATGCGATCAGCATCAAGAACCACATGAATCTCCAGAAAAGAACTCGACATAAATCCTCTATGTATGATGACGAAGATGAAGGTGCCCTAAAACGTCATGCTATTTCTCCATCATTGATAAGACTGCAACGTAACGTTGAGATTTGGGTCAGATGGTTTAACTCAGTGAAAGCCACTGTTTTAACCAATGCTTATGAATTCACAGGTTATGAAGATGAAACACTAGATCTCCTGTTATTGTTCCTTAAGAATTACCTTGAGGATATGCCGTCTAAATGTACAACGGTAGAAAAGATCATATCTGTACTCAACCAGCATAGTTTTCCTGAGAAGGCTGAAGAGAAGGAGGAAAATCTCCAGATTGATGAGGAATGGGCTAAGAACATTTTAGTCAGGCTAGAAAAGACGAAGATTGACTCAGTTGAGGATGTAAAGAAGGTCATAACTGAAGGAGACAAACATGAGCTGGTAGGATACAATCAGTGGTTTCAATACTTGATTAACAACGAACCTCAACACACTACATTTCATGAGAAAATTACGTCTAAACAGCTTTGGGTGTTGATCAAATACATGTCGAATACATGGATAAAAGAGATTCACAAGAAAGGTCGTCACTATAGGAGACTTCAAGATTGGTTGTTCTACATCCTTGTTCATACTCCTGAAAGAGTTACAGCGGAATATACCAGCATTCTACGGGATTTGGGGAAGAAATGCTTAGAGCTTATCCAAAAGAAACCGGTTGAGGCTCATGAGAACAAGATTACACTTCCCAAAGAAATGGCAGAATTGAACGTCGAAATTCCAGCTGCTGTGGAGAATATGACTATCACCGAGTTAACTGTTTCCGTGATAGCCGTGAATTATGGGCAGAA GGATCTCATAGAGtcagagagacctgtt

**SMD1b^Lys^_CDS (C-mod)**

aacaggtctcaggctcaacaATGAAGCTCGTCAGGTTTTTGATGAAATTGAACAACGAAACAGTTTCAATCGAGCTTAAGAACGGAACCATTGTTCATGGAACCATTACTGGTGTAGATGTTAGCATGAACACTCATTTAAAAGCTGTGAAACTCACTCTGAAAGGGAAGAATCCAGTTACATTAGACCACTTAAGTGTCAGGGGAAACAACATTCGCTATTATATTCTTCCGGACAGTTTGAATCTGGAGACTTTGCTGGTTGAAGACACTCCAAGAATCAAACCCAAAAAGCCAACTGCAGGTAAGATTCCAGCAGGTAAAGGAAAAGGTAAAGGAAAAGGTAAAGGAAAAGGCAAAGGTGGTAAATAATGAtcagagagacctgtt

**gSMD1b^Lys^_genomic (C-mod)**

aacaggtctcaggctcaacaATGAAGCTCGTCAGGTATGGTTTCTACTCGGATTCTCTTTATTTCTTCGGTTTAATTGGAATTTGCTTCGAAATTTTGAGATTTCTCTTTGTTTTCATGCTCTTCGATGTTTGATTTCGTATCTGGATTTTTTCGCAGGTTTTTGATGAAATTGAACAACGAAACAGTTTCAATCGAGCTTAAGAACGGAACCATTGTTCATGGAACCATTACTGGTACGCTTCTCTTAAAAACCCAGCTCTATTCGCTGTTTAGGGTTTTGTGTAAAATCTACGATGCTTACCTGTAATCGATGGTTACTCGCGTATTCACAAATTCTGATGGTGTAGTTTGAGTTTAGCTAGTGTCTTTCCCCTAATAATGCTTTGTTGGTATTAACCCTAGTGACACTGGTCAAGTCAGATGCAGATGAGTACACTAGATTTTACTTGAATCGAGGTTTATGAGATAGTTTAGTTCTTCTTCAAACTTTGATCACCCATGAGGTAGTTTACTTTCTCCTTTGATGGTTGTAACCAATGGAGCTCTCGAATTTACTAATTCTGATGTGTAATTCTGTTAATAGACCTAGAAATGCATTGTGGGTGTTGAATCTCTCTTGTTTGCAATAAGTGAATGATAATGGTGGTCTAGTCAGATGGAGATGATACATTTTATTTTGCTTTATAGTTGGGTGTATGAGAATGCTTAGTTCTTCTGGGAGTGAGTTTAGCACATGATTATATATGAGATAGTTTACTTTAGTTTCCCTCTTTATCATTGTGGACCTCTCTTGTTAACAAATCCTGAGGCTTAATTCTGTTATTAGTGATAGTATCTGAAGAAGTTAGAGTTTAGCTGAGTCTTAATTATTTTTTTACCTCGAAATGCTTTGTTGGTAACCCTCTTGTTAGCAAGAACTGAGTGATACTTGTCGAGTCAGATGCAGATGATTAGATTTAGCACATGAAAGTCACTCTTATCTCTTTCCTTGTAAAGAAAATCTCTGATTTTTCACAGAACAGTTACCGTGTGGCTTCTTGTTTCAACTTCTCTTTTAGTCTGATTGATCTAATACAAGTGGTCTCTGCaGTGAGACCTGTTGCAAAATGCGCtGTTAATGTTTGGTTTGGTTTCCTTTTTCTGCCATCATCTTGTTTAGAATGCAATTATCAATCAACTCACTGACTGGTACTATCTACTTGTGATGACTTAATGCAGGTGTAGATGTTAGCATGAACACTCATTTAAAAGCTGTGAAACTCACTCTGAAAGGGAAGAATCCAGTTACATTAGACCACTTAAGTGTCAGGGGAAACAACATTCGCTATTATATTCTTCCGGACAGTTTGAATCTGGAGACTTTGCTGGTTGAAGACACTCCAAGAATCAAACCCAAAAAGCCAACTGCAGGTAACAAAACATTTCTTTTTTCTGAACTATGCCTTTGTTTTTGTTTTTGTTTTTGCTTAGACTCATCTTTCTATTGTTCTCTTTGGTATCAGGTAAGATTCCAGCAGGTAAAGGAAAAGGTAAAGGAAAAGGTAAAGGAAAAGGCAAAGGTGGTAAtcagagagacctgtt

**Supplemental Table S6. List of plasmids. GreenGate entry and destination vectors (Lampropoulos *et al.* 2013) were used in this study to generate the final constructs.**

| **Plasmid ID** | **Promoter**  **(A-mod)** | **N-tag**  **(B-mod)** | **Insert (C-mod)** | **C-tag**  **(D-mod)** | **Terminator**  **(E-mod)** | **Selection markers (Bacteria/Plants)** | **Backbone** |
| --- | --- | --- | --- | --- | --- | --- | --- |
| *pAD-GEMIN2* |  |  | GEMIN2 cds |  |  | Ampicilin | pVZ22 ^1)^ |
| *pBD-GEMIN2* |  |  | GEMIN2 cds |  |  | Kanamycin | pVZ23 ^2)^ |
| *pBD-SPF30* |  |  | SPF30 cds |  |  | Ampicilin | pVZ23 ^2)^ |
| *pAD-SMEa* |  |  | SMEa cds |  |  | Ampicilin | pVZ22 ^1)^ |
| *pBD-SMEa* |  |  | SMEa cds |  |  | Kanamycin | pVZ23 ^2)^ |
| *pAD-SMFa* |  |  | SMFa cds |  |  | Ampicilin | pVZ22 ^1)^ |
| *pBD-SMFa* |  |  | SMFa cds |  |  | Kanamycin | pVZ23 ^2)^ |
| *pAD-SMGa* |  |  | SMGa cds |  |  | Ampicilin | pVZ22 ^1)^ |
| *pBD-SMGa* |  |  | SMGa cds |  |  | Kanamycin | pVZ23 ^2)^ |
| *pAD-SMGb* |  |  | SMGb cds |  |  | Ampicilin | pVZ22 ^1)^ |
| *pBD-SMGb* |  |  | SMGb cds |  |  | Kanamycin | pVZ23 ^2)^ |
| *pAD-SMD1a* |  |  | SMD1a cds |  |  | Ampicilin | pVZ22 ^1)^ |
| *pBD-SMD1a* |  |  | SMD1a cds |  |  | Kanamycin | pVZ23 ^2)^ |
| *pAD-SMD1b* |  |  | SMD1b cds |  |  | Ampicilin | pVZ22 ^1)^ |
| *pBD-SMD1b* |  |  | SMD1b cds |  |  | Kanamycin | pVZ23 ^2)^ |
| *pAD-SMD2a* |  |  | SMD2a cds |  |  | Ampicilin | pVZ22 ^1)^ |
| *pBD-SMD2a* |  |  | SMD2a cds |  |  | Kanamycin | pVZ23 ^2)^ |
| *pAD-SMD2b* |  |  | SMD2b cds |  |  | Ampicilin | pVZ22 ^1)^ |
| *pBD-SMD2b* |  |  | SMD2b cds |  |  | Kanamycin | pVZ23 ^2)^ |
| *pAD-PRMT5* |  |  | PRMT5 cds |  |  | Ampicilin | pVZ22 ^1)^ |
| *pBD-PRMT5* |  |  | PRMT5 cds |  |  | Kanamycin | pVZ23 ^2)^ |
| *pAD-PICLN* |  |  | PICLN cds |  |  | Ampicilin | pVZ22 ^1)^ |
| *pBD-PICLN* |  |  | PICLN cds |  |  | Kanamycin | pVZ23 ^2)^ |
| *pAD-PRMT7* |  |  | PRMT7 cds |  |  | Ampicilin | pVZ22 ^1)^ |
| *pBD-PRMT7* |  |  | PRMT7 cds |  |  | Kanamycin | pVZ23 ^2)^ |
| *pAD-ASIL1* |  |  | ASIL1 cds |  |  | Ampicilin | pVZ22 ^1)^ |
| *pAD-SMD1bΔC* |  |  | SMD1bΔC cds |  |  | Ampicilin | pVZ22 ^1)^ |
| *pBD-SMD1bΔC* |  |  | SMD1bΔC cds |  |  | Kanamycin | pVZ23 ^2)^ |
| *pAD-SMD1b^Lys^* |  |  | SMD1b^Lys^ cds |  |  | Ampicilin | pVZ22 ^1)^ |
| *pBD-SMD1b^Lys^* |  |  | SMD1b^Lys^ cds |  |  | Kanamycin | pVZ23 ^2)^ |
| *pGEMIN2::GEMIN2CDS* | pGEMIN2 | B-dummy | GEMIN2 cds | D-dummy | tRBCS | Spec/Basta | pGGZ003 |
| *pGEMIN2::HsGEMIN2* | pGEMIN2 | B-dummy | HsGEMIN2 cds | D-dummy | tRBCS | Spec/Basta | pGGZ003 |
| *pGEMIN2::ScBRR1P* | pGEMIN2 | B-dummy | ScBRR1P cds | D-dummy | tRBCS | Spec/Basta | pGGZ003 |
| *p35S::SMD1bCDS* | p35S | B-dummy | SMD1b cds | D-dummy | tRBCS | Spec/Basta | pGGZ003 |
| *p35S::SMD1b^Lys^CDS* | p35S | B-dummy | SMD1b^Lys^ cds | D-dummy | tRBCS | Spec/Basta | pGGZ003 |
| *pSMD1b::SMD1bCDS::tSMD1b* | pSMD1b | B-dummy | SMD1b cds | D-dummy | tSMD1b | Spec/Basta | pGGZ003 |
| *pSMD1b::SMD1b^Lys^CDS::tSMD1b* | pSMD1b | B-dummy | SMD1b^Lys^ cds | D-dummy | tSMD1b | Spec/Basta | pGGZ003 |
| *pSMD1b::gSMD1b::tSMD1b* | pSMD1b | B-dummy | gSMD1b | D-dummy | tSMD1b | Spec/Basta | pGGZ003 |
| *pSMD1b::gSMD1b^Lys^::tSMD1b* | pSMD1b | B-dummy | gSMD1b^Lys^ | D-dummy | tSMD1b | Spec/Basta | pGGZ003 |
| *pGEMIN2::GFP-GEMIN2 CDS* | pGEMIN2 | GFP | GEMIN2 cds | D-dummy | tRBCS | Spec/Basta | pGGZ003 |
| *pGEMIN2::GFP-HsGEMIN2 CDS* | pGEMIN2 | GFP | HsGEMIN2 cds | D-dummy | tRBCS | Spec/Basta | pGGZ003 |
| *pGEMIN2::GFP-ScBRR1P CDS* | pGEMIN2 | GFP | ScBRR1P cds | D-dummy | tRBCS | Spec/Basta | pGGZ003 |
| *p35S::N-Citrine_GEMIN2* | p35S | N-Citrine | GEMIN2 cds | D-dummy | tRBCS | Spec/Basta | pGGZ003 |
| *p35S::N-Citrine_ASIL1* | p35S | N-Citrine | ASIL1 cds | D-dummy | tRBCS | Spec/Basta | pGGZ003 |
| *p35S::C-Citrine_SPF30* | p35S | C-Citrine | SPF30 cds | D-dummy | tRBCS | Spec/Basta | pGGZ003 |
| *p35S::C-Citrine_SmGb* | p35S | C-Citrine | SmGb cds | D-dummy | tRBCS | Spec/Basta | pGGZ003 |

Footnotes: 1) PVZ22 is pGADT7 derivative, modified for GreenGate; 2) PVZ23 is pGBKT7 derivative, modified for GreenGate.
